# Novel Route to Produce Hydrocarbons from Woody Biomass Using Molten Salts

**DOI:** 10.1021/acs.energyfuels.2c02044

**Published:** 2022-10-11

**Authors:** Balaji Sridharan, Homer C. Genuino, Daniela Jardan, Erwin Wilbers, Henk H. van de Bovenkamp, Jozef G. M. Winkelman, Robbie H. Venderbosch, Hero J. Heeres

**Affiliations:** † Department of Chemical Engineering, Engineering and Technology Institute Groningen (ENTEG), University of Groningen, Nijenborgh 4, 9747 AGGroningen, Netherlands; ‡ Biomass Technology Group B.V., Josink Esweg 34, 7545 PNEnschede, Netherlands

## Abstract

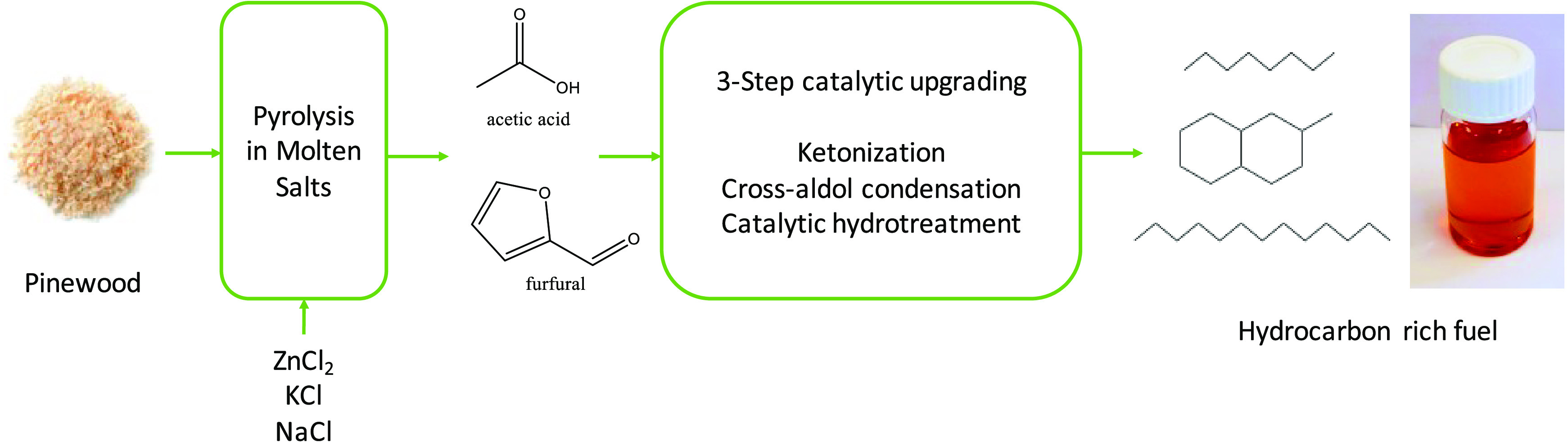

The thermochemical decomposition of woody biomass has been widely identified as a promising route to produce renewable biofuels. More recently, the use of molten salts in combination with pyrolysis has gathered increased interest. The molten salts may act as a solvent, a heat transfer medium, and possibly also a catalyst. In this study, we report experimental studies on a process to convert woody biomass to a liquid hydrocarbon product with a very low oxygen content using molten salt pyrolysis (350–450 °C and atmospheric pressure) followed by subsequent catalytic conversions of the liquids obtained by pyrolysis. Pyrolysis of woody biomass in molten salt (ZnCl_2_/NaCl/KCl with a molar composition of 60:20:20) resulted in a liquid yield of 46 wt % at a temperature of 450 °C and a molten salt/biomass ratio of 10:1 (mass). The liquids are highly enriched in furfural (13 wt %) and acetic acid (14 wt %). To reduce complexity and experimental issues related to the production of sufficient amounts of pyrolysis oils for further catalytic upgrading, model studies were performed to convert both compounds to hydrocarbons using a three-step catalytic approach, viz., (i) ketonization of acetic acid to acetone, (ii) cross-aldol condensation between acetone and furfural to C_8_–C_13_ products, followed by (iii) a two-stage catalytic hydrotreatment of the latter to liquid hydrocarbons. Ketonization of acetic acid to acetone was studied in a continuous setup over a ceria–zirconia-based catalyst at 250 °C. The catalyst showed no signs of deactivation over a period of 230 h while also achieving high selectivity toward acetone. Furfural was shown to have a negative effect on the catalyst performance, and as such, a separation step is required after pyrolysis to obtain an acetic-acid-enriched fraction. The cross-aldol condensation reaction between acetone and furfural was studied in a batch using a commercial Mg/Al hydrotalcite as the catalyst. Furfural was quantitatively converted with over 90% molar selectivity toward condensed products with a carbon number between C_8_ and C_13_. The two-stage hydrotreatment of the condensed product consisted of a stabilization step using a Ni-based Picula catalyst and a further deep hydrotreatment over a NiMo catalyst, in both batch setups. The final product with a residual 1.5 wt % O is rich in (cyclo)alkanes and aromatic hydrocarbons. The overall carbon yield for the four-step approach, from pinewood biomass to middle distillates, is 21%, assuming that separation of furfural and acetic acid after the pyrolysis step can be performed without losses.

## Introduction

Rapid depletion of traditional fossil energy and rising CO_2_ emissions have highlighted the urgent need for a more sustainable and environmentally friendly source for fuels and chemicals. Among all alternatives identified, lignocellulosic biomass is one of the few promising feedstocks for the production of sustainable carbon-based biofuels. Of the various thermochemical processes available to valorize lignocellulosic biomass, pyrolysis is considered a straightforward, low-cost, and energy-efficient way of converting biomass to liquid fuels.^1,2^

Molten salt pyrolysis of woody biomass has gathered increasing interest recently, especially in small lab-scale experiments. Its ability to dramatically increase the heating rate for the thermal decomposition of biomass makes it very attractive for the pyrolysis of woody biomass.^3,4^ Its catalytic activity to mildly deoxygenate intermediate pyrolysis products to favor the production of monoaromatic hydrocarbons has been reported.^5−7^ Eutectic molten salt mixtures containing zinc chloride in particular have been shown to have a positive effect on product yields and selectivity.^8^ The use of molten salts was shown to improve liquid yields when compared to conventional wood pyrolysis, with the highest liquid yield of 66 wt % reported at temperatures between 350 and 450 °C.^8^ Moreover, the liquid products were shown to be enriched in furfural and acetic acid.^8^

A combination of ketonization and (cross) aldol condensation has been identified as an attractive pathway to convert short-chain biomass-based platform chemicals to long-chain liquid hydrocarbon fuels and chemicals and was also used in this study as a means to convert furfural and acetic acid to hydrocarbons.^9,10^ The ketonization of acetic acid to acetone and CO_2_ is the most widely studied among the organic acids, and transition metal oxides have been used as catalysts. Among them, CeO_2_,^11−13^ TiO_2_,^14−17^ ZrO_2_,^18−21^ and MnO_2_^19^ have been widely studied. The use of bimetallic catalysts, such as Co–Mo/Al_2_O_3_ and ZrMnO_*x*_, has also been studied, and a 88% selectivity to acetone at full conversion of acetic acid was reported.^22,23^ More interestingly, doping of CeO_2_ with Zr was shown to increase the strength and number of both the basicity and acidity, making them very active for the gas-phase ketonization of pentanoic acid.^24^

The subsequent cross-aldol condensation of acetone and furfural is an efficient approach to increase the carbon number of the products and has been widely reported in the literature for a range of aldehydes and ketones. Some examples are the synthesis of sustainable jet fuels from carbohydrate-derived dehydration products, such as furfural and 5-hydroxymethylfurfural.^25^ Both homogeneous basic catalysts, such as NaOH,^10,26^,^27^ and heterogeneous transition metal oxides composed of Zr, Pd, Mg, and Al have been widely used.^28−32^ For the combination of acetone–furfural, a maximum selectivity of 90% has been reported for C_8_ and C_13_ products using a Mg–Al mixed oxide catalyst system.^28^ Bifunctional heterogeneous catalysts (Mg–Al and Mg–Zr) on zeolite and hydrotalcite supports in particular have also shown high promise and are reported to have a high selectivity (86%) for the C_8_ cross aldol product.^33,34^ However, rapid catalyst deactivation as a result of coking is a major issue.

The C_8_ and C_13_ products from furfural–acetone cross-aldol condensation reactions may be converted to hydrocarbons in the middle-distillate range by a catalytic hydrodeoxygenation step. However, hydrodeoxygenation of the furfural–acetone cross-aldol products is complicated mainly because of the different reactive functional units (C=O, C–OH, and C–O–C) in the starting feed.^35,36^ Although hydrodeoxygenation of aldol condensation products has been reported using bifunctional noble metal catalysts, a common consensus within the literature is to use a two-step approach involving a low-temperature catalytic hydrogenation step prior to the deoxygenation step at elevated temperatures.^27,37−39^ With this approach, reactive aldehydes/ketones are converted, at low temperatures, to less reactive alcohols, thereby limiting coke formation at elevated deoxygenation temperatures. Noble metal catalysts (Pd, Pt, Ru, Ni, and Cu) have been widely studied for the low-temperature hydrogenation step, and Pt on an acidic alumina support was shown to be the most suitable noble metal catalyst.^40−43^ For the deep hydrodeoxygenation to hydrocarbons, harsh reaction conditions are required, and this makes removal of bound oxygen to levels below 1% very challenging.

**Figure 1:**
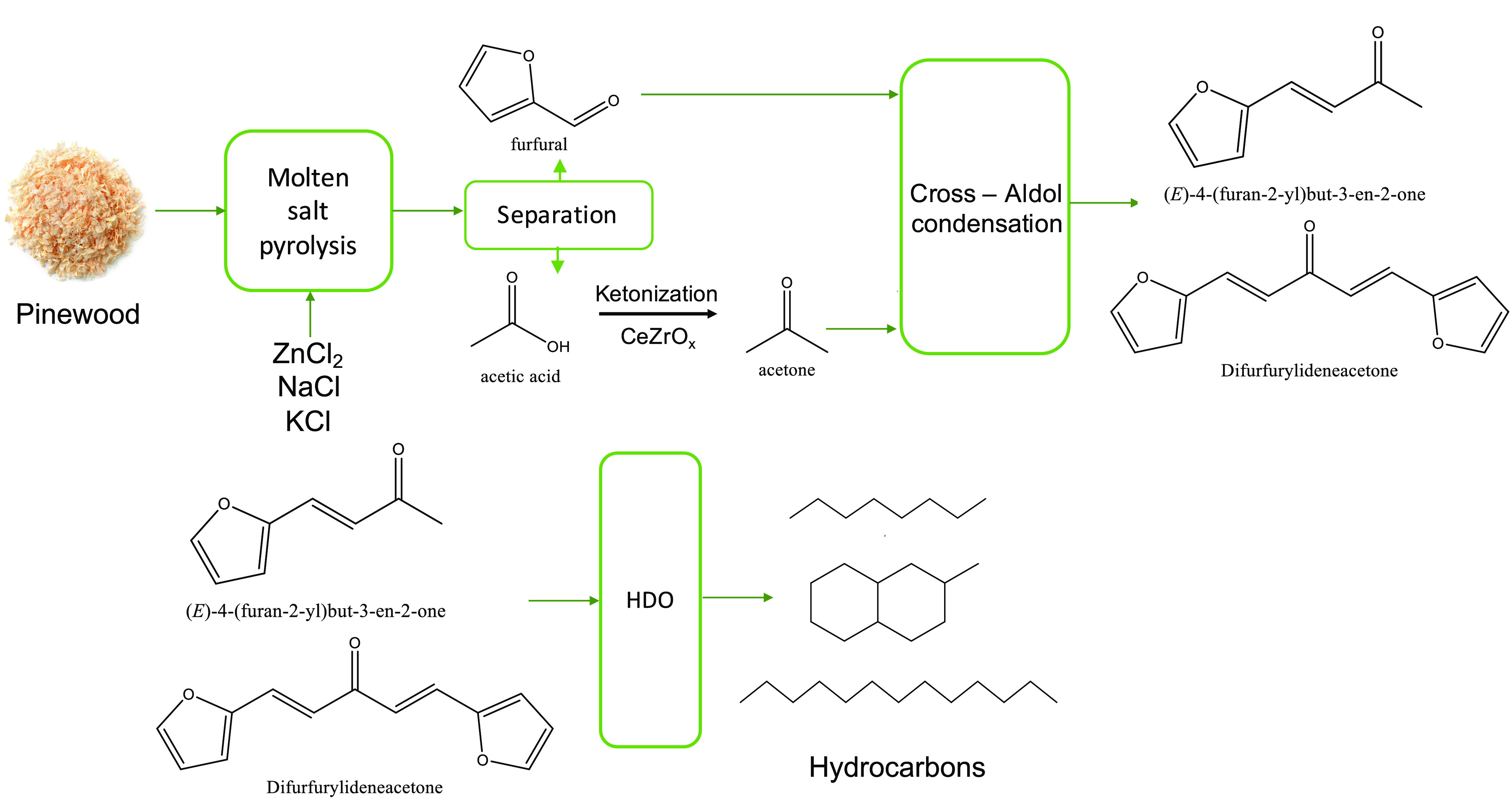
Overall concept of the current study to obtain hydrocarbons from pinewood biomass.

In this study, we report a process to convert pinewood biomass to a hydrocarbon-rich product with a very low oxygen content by an integrated approach involving molten salt pyrolysis (350–450 °C and atmospheric pressure), followed by catalytic conversions of acetic acid and furfural present in the pyrolysis liquid. A schematic representation of the concept is shown in Figure 1. A eutectic mixture of ZnCl_2_, NaCl, and KCl was used as the molten salt to produce a pyrolysis liquid product rich in furfural and acetic acid. Acetic acid was subsequently converted to acetone by ketonization using a CeZrO_*x*_ catalyst in a continuous packed bed setup. Separation of acetic acid and furfural is shown to improve catalyst performance, particularly in the ketonization step of acetic acid, where the presence of furfural causes a negative effect on the catalyst activity and stability. Cross-aldol condensation of furfural and acetone obtained by ketonization was performed using commercially available Mg–Al hydrotalcite catalysts in batch, and the yield of the desired C_13_ product was optimized. The furfural–acetone condensation products were then deoxygenated using a two-step catalytic hydrotreatment process: (i) stabilization using a Ni-type Picula catalyst followed by (ii) a deep hydrodeoxygenation step using a NiMo/Al_2_O_3_ catalyst. Although the individual process steps in this concept (ketonization, aldol condensation, and catalytic hydrotreatment) are known, the integration and determination of overall carbon yields are a novelty of this paper.

## Experimental Section

### Chemicals

Powdered pinewood was supplied by Aston University, U.K., and its elemental composition is shown in Table S1 of the Supporting Information. Acetic acid (99%), acetone (99%), furfural (99%), and *n*-butyl ether (internal standard, 98%) were purchased from Sigma-Aldrich. The inorganic salts ZnCl_2_, KCl, and NaCl used were of analytical grade and sourced from Sigma-Aldrich. The individual salts were pre-dried by heating in an oven at 350 °C overnight. After cooling, the salts were crushed into fine particles, weighed, mixed at the required ratio, and stored in a vacuum desiccator. An aqueous solution of Ce(NO_3_)_3_·6H_2_O (99%) and ZrO(NO_3_)_2_·*x*H_2_O (99%) for the preparation of the ketonization catalysts was purchased from Sigma-Aldrich. Hydrotalcite catalysts for the aldol condensation reaction were supplied by Kisuma Chemicals. Hydrotreatment catalysts (NiMo and Picula) were supplied by the Biomass Technology Group (BTG).

### Catalysts

The CeZrO_*x*_ catalyst was synthesized by a co-precipitation method, as described elsewhere with minor modifications.^44^ Briefly, an aqueous solution of Ce(NO_3_)_3_·6H_2_O and ZrO(NO_3_)_2_·*x*H_2_O was prepared. The amounts of the individual precursors were selected in such a way to obtain a Ce/Zr molar ratio of 1. This solution was added to an alkaline solution (pH 10) of NH_4_OH to initiate precipitation. The resulting slurry was aged under stirring at room temperature for 72 h at pH 10. The precipitate was separated by filtration, washed with deionized water and ethanol, dried at 100 °C for 12 h, and then calcined at 450 °C for 2 h in an oven.

The hydrotalcite employed in the study has a Mg/Al ratio of 4.1 with a median particle size of 5.0 μm. The catalyst was calcined at 550 °C for 5 h prior to the cross-aldol condensation experiments.

Details on the preparation of the Picula catalyst are provided in previous publications of our research group.^45−47^ Both the Picula and NiMo catalysts were activated *ex situ* at 350 °C for 3 h with hydrogen.

### Molten Salt Pyrolysis Experiments

Pinewood biomass was pyrolyzed in a small-scale batch reactor (1.0 g of wood intake) with a constant flow of an inert gas (Figures S1 and S2 of the Supporting Information). Typically, 1.0 g of the wood sample was mixed with 10.0 g of ZnCl_2_/KCl/NaCl mixture (eutectic mixture at a 60:20:20 molar ratio) inside a glass insert, which was then placed inside the metal reactor. The reactor was then placed in a hot fluidized sand bath to start the pyrolysis reaction. A constant flow of nitrogen (20 mL/min) was used to transfer the vapors produced during pyrolysis to the condensers (maintained at −40 °C using a liquid nitrogen–ethanol mixture). The mass of condensable bio-oil was determined from the difference in the weight of the tube linings and condensers before and after pyrolysis. The bio-oil was taken from the tube linings and condensers by thoroughly washing with tetrahydrofuran (THF). The non-condensable gases were collected in a gas bag (SKC Tedlar 3 L sample bag, 9.5 × 10 in., with polypropylene septum fitting) and weighed by water displacement. The mass of the solid product (i.e., residues, char, and ash) excluding the salt was determined from the difference of the weight of the glass tube contents before and after pyrolysis. All product yields were reported as weight percent in terms of wood intake on a dry basis and an average of at least two trials.

The water content and composition of the pyrolysis oil (i.e., low-molecular-weight compounds) were determined by Karl Fischer titration and gas chromatography–mass spectroscopy (GC–MS), respectively (see details in the Analytical Techniques section). The yield (on the basis of dry feed intake) of one of the main products in the product oil, furfural, was quantified by gas chromatography–mass spectroscopy with a flame ionization detector (GC–MS–FID) (eq 1). Acetic acid cannot be quantified accurately using GC–MS–FID analysis as a result of peak tailing and was instead quantified using high-performance liquid chromatography (HPLC) analysis.
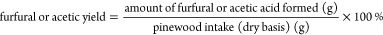
1

### Ketonization Experiments

**Figure 2:**
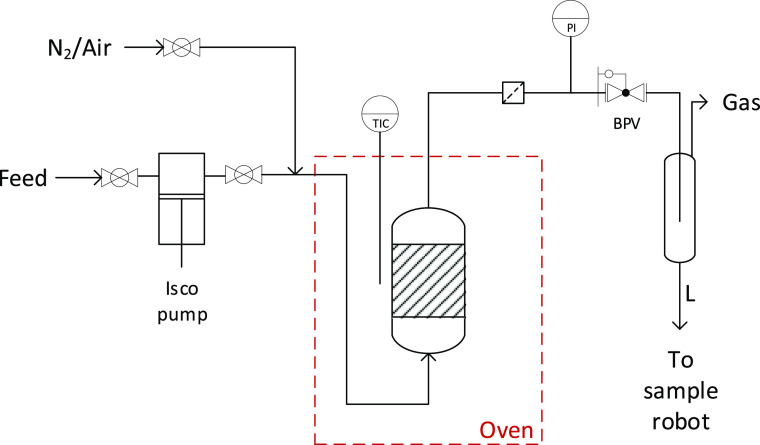
Schematic of the packed bed reactor used for the continuous ketonization experiments.

To reduce complexity and experimental issues related to the production of sufficient amounts of pyrolysis oils, further upgrading of the pyrolysis oil was studied in detail with representative components from the pyrolysis oil. Ketonization of acetic acid was studied in a fixed bed reactor (stainless steel, with an inner diameter of 10 mm). The reactor was placed inside an electrically heated, temperature-controlled furnace (see Figure 2). An aqueous feed of water and acetic acid was fed to the reactor in an upflow configuration by the use of a syringe pump along with a gas flow of nitrogen, which was controlled by a mass flow controller. The reaction pressure of ketonization was controlled by a back-pressure valve located downstream of the reactor. The products were collected in 4 mL glass vials using an autosampler, constructed in-house, whose collection frequency could be varied. The collected products were then weighed, and the amounts of acetone and acetic acid were quantified using HPLC analysis. The yield and selectivity of acetone and conversion of acetic acid are calculated on a carbon basis, as shown in eqs 2, 3, and 4, respectively.acetone yield
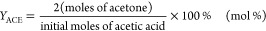
2selectivity of acetone
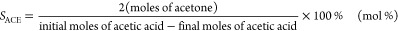
3conversion of acetic acid
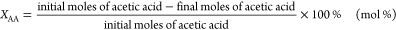
4

### Aldol Condensation Experiments

Cross-aldol condensation experiments with furfural and acetone were carried in a batch setup consisting of a 50 mL stirred Parr autoclave connected to a Parr 4843 model controller. The reactor was loaded with 17 g of an organic mixture of furfural, acetone, and a certain mass of catalyst. The reactor was pressured with nitrogen to a set reaction pressure of 30 bar. The reactor was then heated to the desired temperature. The liquid products after the reaction were separated from the solid products and the spent catalysts through centrifugation. The solid products were washed thoroughly with acetone, dried overnight at 60 °C, and weighed to determine their mass yield. The concentration of the individual compounds in the product liquid was measured using gas chromatography.

The overall yields of the cross-aldol condensation products (C_8_ and the C_13_), along with the conversion of furfural, were optimized by design of experiments (DOE). The definitions of the four responses are shown in eqs 5–8. The composition of the organic mixture, catalyst loading, batch time, and reaction temperature were varied in this study. Design Expert (version 12) software was used for the DOE generation and analysis using a quadratic randomized response surface methodology (RSM) with two repeating points. The levels of the four variables used in the experimental design are shown in Table 1. The output data were modeled using a quadratic fit. Details on the model-fitting procedure and relevant statistical information are given in Table S3 of the Supporting Information.furfural conversion
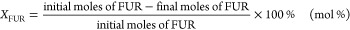
5C_8_ monomer molar yield
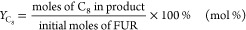
6C_13_ dimer molar yield
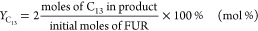
7solid yield
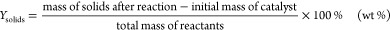
8

**Table 1:** Levels of the Four Variables for the Cross-Aldol Condensation Reaction of Acetone and Furfural

factor	name	unit	minimum	maximum
A	catalyst loading	wt %	2.5	15
B	furfural/acetone ratio	mol/mol	0.5	2.5
C	temperature	°C	50	120
D	reaction time	h	0.2	24

### Catalytic Hydrotreatment Experiments

A two-step catalytic hydrotreatment was employed, first with Picula followed by a second step with an unsulfided NiMo catalyst. The process conditions for the two hydrodeoxygenation (HDO) reactions are shown in Table 2.

**Table 2:** Process Conditions for the Hydrodeoxygenation Experiments Performed

process variable	HDO 1: Picula	HDO 2: NiMo
temperature	175 °C for 1 h and 275 °C for 3 h	350 °C for 4 h
pressure	100 bar	150 bar

Batch hydrodeoxygenation experiments were performed in a 100 mL Parr reactor. A total of 25 g of product mixture from the cross-aldol condensation experiments and 2 g of catalyst were added to the reactor. The reactor was flushed thrice with hydrogen (10 bar) to remove any residual air. The reactor was pressure-tested at 200 bar with pressurized hydrogen prior to an experiment and then set to the desired pressure. The reactor was subsequently heated to the intended temperature. After the reaction, the reactor was cooled, gas samples were collected using a 100 mL syringe, and the composition was determined using gas chromatography with a thermal conductivity detector (GC–TCD) analysis. The mass of the reactor containing the products and the stirrer were measured, and the entire content inside the reactor was emptied into a centrifuge vial. The aqueous phase, the organic phase, and the catalysts were separated using centrifugation (4500 rpm for 15 min), and each of the three phases was weighed. The composition of the organic phase was measured using gas chromatography. The sedimented solids were washed thoroughly with acetone, dried overnight, and weighed. The char yield was calculated from the difference in the mass of the solids and the mass of catalysts added. The mass of the gas phase was calculated by difference.

### Analytical Techniques

#### Water Content

The water content of the products was measured by Karl Fischer titration using a Metrohm MRD 296 with 702 SM Titrino and 703 Ti stand, following the ASTM E203 standard procedure. About 0.010 g of sample was injected in an isolated glass chamber containing Hydranal Karl Fischer solvent, and the titrations were carried out using the Karl Fischer titrant Composit 5K. Triplicate measurements for each sample were conducted, and the average value is reported.

#### GC–MS

GC–MS analyses were performed on a Hewlett-Packard (HP 6890 series GC system) gas chromatograph equipped with a RXI-5Sil-MS capillary column (30 m × 0.25 mm inner diameter and 0.25 μm film thickness) and a quadrupole Hewlett-Packard 6890 mass selective detector attached. Helium was used as a carrier gas at a flow rate of 2 mL min^–1^. The injector was set at 280 °C. The oven temperature was kept at 40 °C for 5 min, then increased to 280 °C at a rate of 3 °C min^–1^, and held at 280 °C for 15 min.

#### GC–MS–FID

For the identification of individual components in the lignin oil, gas chromatography analyses were performed using a Hewlett-Packard 5890 GC provided with a FID, coupled with a quadruple Hewlett-Packard 6890 MSD (GC–MS–FID). The GC column was a RTX-1701 (60 m × 0.25 mm inner diameter and 0.25 μm film thickness). The sample to be measured was diluted at a 1:10 ratio in tetrahydrofuran (THF), and then di-*n*-butyl ether (DBE) was added to serve as an internal standard.

#### Two-Dimensional Gas Chromatography with Time of Flight Mass Spectrometry (GC × GC/TOF-MS)

GC × GC/TOF-MS analysis was performed on an Agilent 7890B system equipped with a JEOL AccuTOF GCv 4G detector and two capillary columns, i.e., a RTX-1701 capillary column (30 m × 0.25 mm inner diameter and 0.25 μm film thickness) connected by a solid-state modulator (Da Vinci DVLS GC2) to a Rxi-5Sil MS column (120 cm × 0.10 mm inner diameter and 0.10 μm film thickness).

#### HPLC

HPLC analysis used for the identification and quantification of acetic acid was performed using a HPLC consisting of an Agilent 1200 pump, a Bio-Rad organic acid column Aminex HPX-87H, a Waters 410 differential refractive index detector, and an ultraviolet (UV) detector. Aqueous sulfuric acid (5 mM) at a flow rate of 0.55 mL/min was used as the mobile phase. The HPLC column was operated at 60 °C. Calibration curves for acetone and acetic acid were prepared for accurate quantification and were based on a minimum of 4 data points with a linear fit of *R*^2^ > 0.99. Samples to be measured were diluted at least 10 times with ultrapure water and filtered with a 0.2 μm syringe prior to analysis.

#### ^13^C Nuclear Magnetic Resonance (NMR)

^13^C NMR spectra were recorded on a Bruker NMR spectrometer (600 MHz) using a 90° pulse and an inverse-gated decoupling sequence with a relaxation delay of 10 s, sweep width of 225 ppm, and 1024 scans. Samples were prepared by dissolving about 100 mg of product in deuterated chloroform (CDCl_3_-*d*_1_, Sigma-Aldrich, 99.5 atom % D).

#### Fourier Transform Infrared Spectroscopy (FTIR)

An attenuated total reflection infrared (ATR-IR) spectrometer was used. Approximately 1 or 2 drops of sample were placed on the sample unit (Graseby Specac Golden Gate with a diamond top), and the infrared (IR) spectra were obtained using a Shimadzu IRTracer-100 FTIR spectrometer with resolution of 4 cm^–1^ and 64 scans.

#### Gas-Phase Analyses

Gas-phase analyses were performed on GC–TCD [Hewlett-Packard 5890 Series II GC equipped with a Poraplot Q Al_2_O_3_/Na_2_SO_4_ column and a molecular sieve (5 Å) column]. The injector temperature was set at 150 °C, and the detector temperature was set at 90 °C. The oven temperature was kept at 40 °C for 2 min, then heated to 90 °C at 20 °C min^–1^, and kept at this temperature for 2 min. A reference gas containing H_2_ (55.19%), CH_4_ (19.70%), CO_2_ (18.01%), CO (3.00%), propane (1.50%), ethane (1.49%), ethylene (0.51%), and propylene (0.51%) was used for quantitative analysis.

#### Elemental Analyses (C, H, N, and S)

Elemental analyses were performed using an EuroVector EA3400 Series CHNS-O analyzer with acetanilide as the reference. The oxygen content was determined indirectly by difference. All analyses were conducted in duplicate, and the average value is reported.

## Results and Discussion

### Molten Salt Pyrolysis of Pinewood

**Figure 3:**
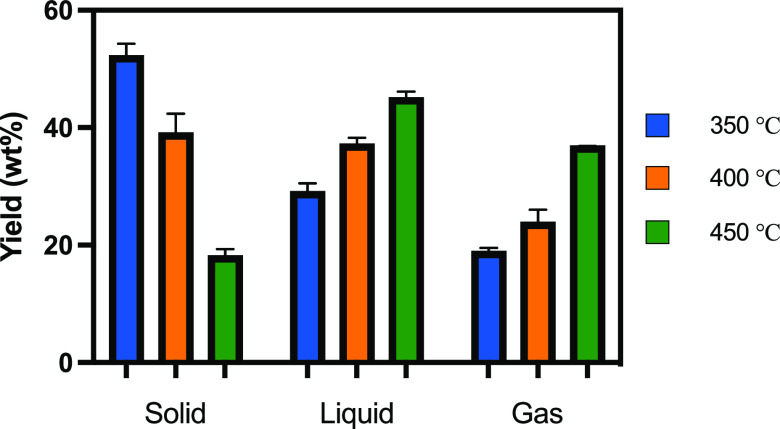
Individual product yields from the pyrolysis of pinewood in molten salts at three different temperatures.

Pyrolysis of pinewood powder in molten salt (ZnCl_2_/KCl/NaCl, 60:20:20 mol ratio) was performed in a batch reactor at gram scale, which was rapidly heated by placing it in a fluidized sand bath at the start of the reaction. The effect of the temperature on the overall product yields is shown in Figure 3. A maximum liquid yield of 45 wt % was measured at a pyrolysis temperature of 450 °C, along with a char yield of 18 wt % and a gas yield of 37 wt %. The mass of non-condensable gases is also dependent upon the pyrolysis temperature and increases from 19 wt % at 350 °C to 37 wt % at 450 °C. Separation of the molten salt and the char after reaction (at temperatures above the melting point of the salt) is possible as a result of their immiscibility and large differences in the density between the char (low density) and molten salts (high density).

**Figure 4:**
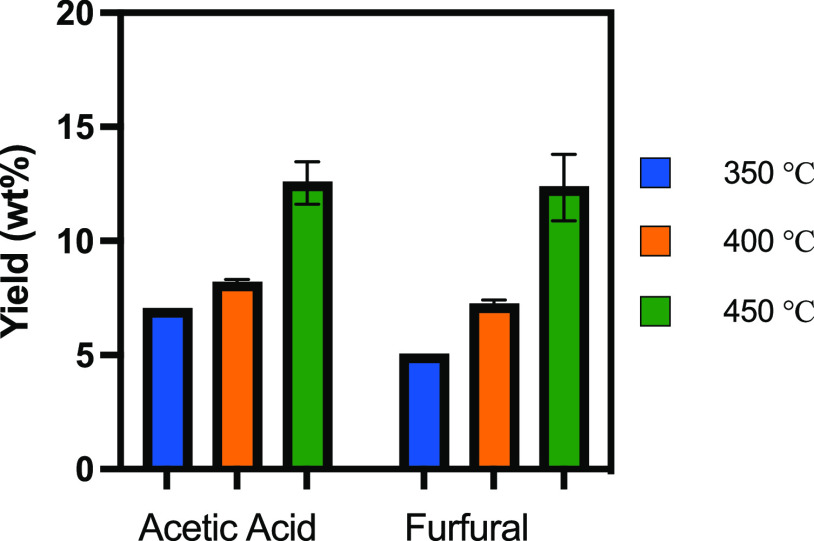
Yields of furfural and acetic acid from the pyrolysis of pinewood in molten salts.

The liquid products from the molten salt pyrolysis of wood showed the presence of significant amounts of acetic acid and furfural (GC–MS). The individual product yields of furfural and acetic acid are shown in Figure 4 at different pyrolysis temperatures. The highest yields of acetic acid (14 wt %) and furfural (15 wt %) were obtained at 450 °C. This high selectivity is in sharp contrast with that typically found in pyrolysis oils from conventional fast pyrolysis of pinewood without molten salts. Conventional fast pyrolysis oil exhibits a very diverse product portfolio, with a large fraction of aldehydes/ketones (e.g., hydroxyacetaldehyde) phenolics (guaiacols), sugars (levoglucosan), and organic acids,^1^ and as such, the high selectivity to furfural and acetic acid is due to the presence of the molten salts.^8^ Although the liquid yields from wood pyrolysis in molten salts, as shown in Figure 3, are lower than the yields typically found for conventional fast pyrolysis (between 60 and 70 wt %), the selective production of furfural and acetic acid could be of potential interest.^1^ Thus far, no sound explanations could be found in the literature for these low yields. Speculatively, it is well possible that the lignin fraction in the wood is actually not converted to low-molecular-weight components, like phenols (visible in GC–MS at very low amounts), but rather end up in the char. The pyrolysis oil also contained small amounts of lighter oxygenated compounds, such as formic acid (see Figures S3 and S4 of the Supporting Information).

### Ketonization of Acetic Acid

The next catalytic steps of the integrated approach (ketonization, cross-aldol condensation, and hydrodeoxygenation) are preferably carried out using the oil obtained after pyrolysis of wood in molten salt. However, this proved challenging as a result of experimental constraints. We were not able to obtain sufficient amounts of pyrolysis oil in the experimental setup for the further catalytic upgrading steps in continuous setups operated at hundreds of hours of time on stream (TOS). As such, it was initially decided to apply model feeds consisting of acetic acid in water for the ketonization step and furfural–acetone mixtures for the cross-condensation step.

**Figure 5:**
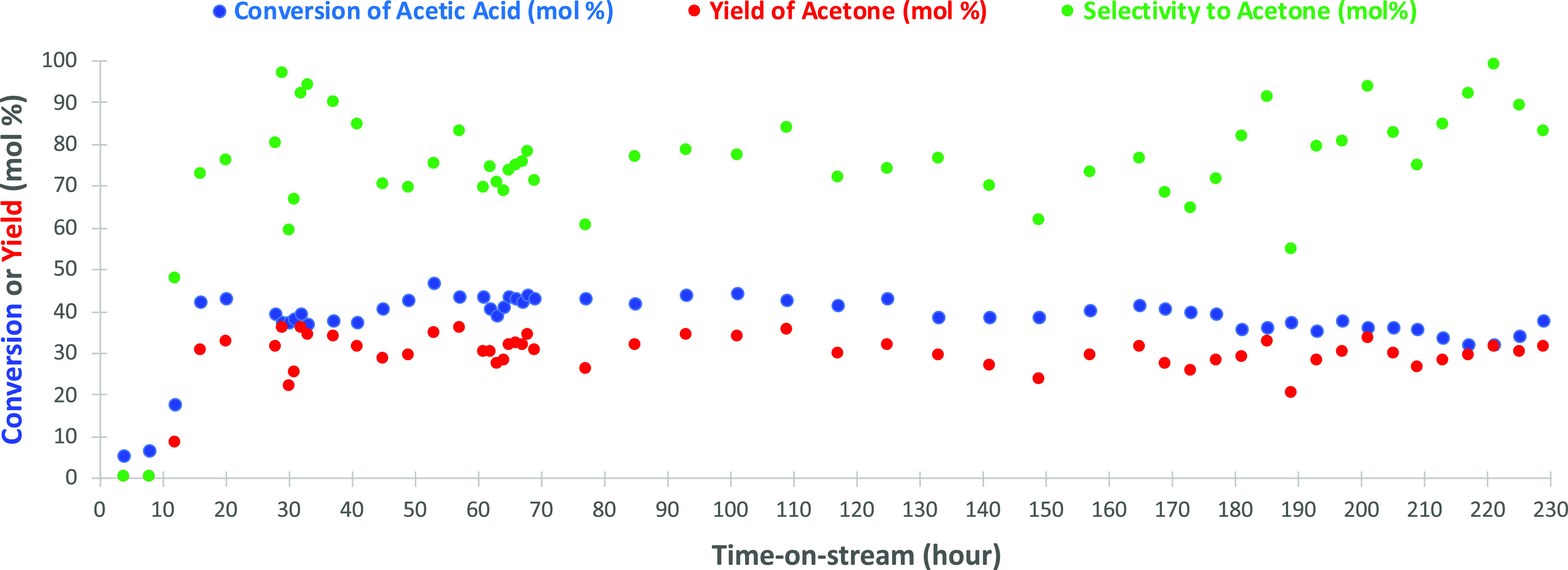
Conversion of acetic acid and the corresponding yield of acetone from the continuous ketonization experiments. Process conditions: 10 wt % acetic acid in water, 2.4 mL h^–1^ nitrogen, 1.0 g of CeZrO_*x*_ catalyst, 250 °C, and 1 atm.

The continuous gas-phase ketonization of acetic acid in water was performed over an activated CeZrO_*x*_ catalyst in a fixed bed reactor (10 wt % acetic acid in water, 2.4 mL h^–1^ nitrogen, 1.0 g of CeZrO_*x*_ catalyst, 250 °C, and 1 atm). The conversion of acetic acid and the selectivity to acetone were measured over a time period of 200+ h to obtain information on the catalyst performance and particularly the long-term stability and product selectivity. The conversion of acetic acid and the yield of acetone over time are shown in Figure 5. The conversion was deliberately set at about 50% at the start to determine catalyst stability, which was shown to be constant over the runtime, indicating high stability of the catalyst. Complete conversion is well possible by tuning process conditions. HPLC analysis of the products shows only acetone as the identifiable compound, hinting at little or no side reactions. The selectivity of acetone is relatively stable and between 70 and 100 mol % over the runtime of 230+ h. The variation is mainly attributed to vaporization of acetone during sample collection. At a higher reaction temperature of 300 °C, 100% conversion of acetic acid was observed with a high selectivity toward acetone (shown in Figure S5 of the Supporting Information).

The catalytic ketonization experiments were carried out using acetic acid in water and not with a representative pyrolysis oil after molten salt pyrolysis. The latter also contains significant amounts of furfural, some minor amounts of formic acid, and trace amounts of phenolics. The effect of the presence of furfural on the conversion of acetic acid was investigated by performing a ketonization experiment with mixtures of acetic acid and furfural in water. This involved a run in the continuous setup with an initial mixture of acetic acid in water, followed by switching to a mixture of acetic acid and furfural in water after 70 h TOS. This resulted in a significant drop in acetic acid conversion from about 45 to 30% (Figure S6 of the Supporting Information), indicating a detrimental effect of furfural on the catalyst performance, possibly as a result of competitive adsorption of furfural on the active sites of the catalyst. After 200 h TOS, the conversion dropped to about 20%, indicating also some catalyst deactivation. These findings imply that it is better to perform the ketonization experiments of acetic acid in the absence of furfural, from both a catalyst activity and stability point of view. As such, it is proposed that, in the overall concept from wood to middle distillates, the pyrolysis oil after molten salt pyrolysis is separated in two fractions, an acetic-acid-rich fraction and a furfural-rich fraction, e.g., by (vacuum) distillation and that the subsequent catalytic steps are performed with these fractions.

### Cross-Aldol Condensation of Furfural and Acetone

Cross-aldol condensation experiments of furfural and acetone were performed in a pressured autoclave in the absence of a solvent, and the yield of the desired C_13_ product was optimized using a factorial-based DOE. The following ranges of process conditions were applied: temperature, 50–120 °C; catalyst loading, 2.50–15 wt %; furfural/acetone ratio, 0.5–2.5 (mol/mol); and batch times, between 0.2 and 24 h. The list of experiments performed as part of the DOE and the corresponding C_13_ yield, furfural conversion, and solid mass yield are given in Table S2 of the Supporting Information. More details on the statistical modeling and data regression methodology are discussed in detail in the Supporting Information.

**Figure 6:**
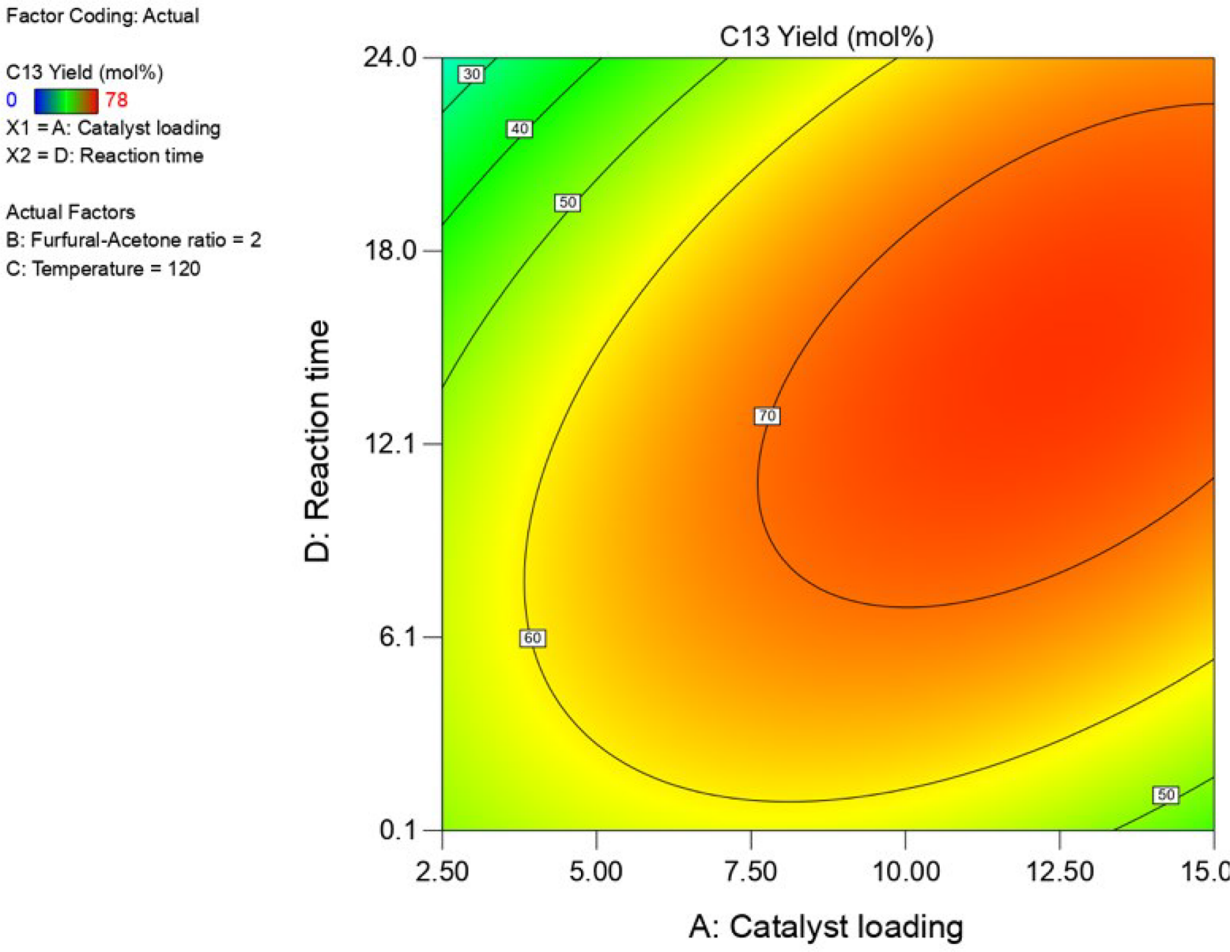
Contour plot showing the C_13_ molar yield versus the catalyst loading and the reaction time at a constant reaction temperature of 120 °C and a furfural/acetone ratio of 1.95 (mol/mol).

**Figure 7:**
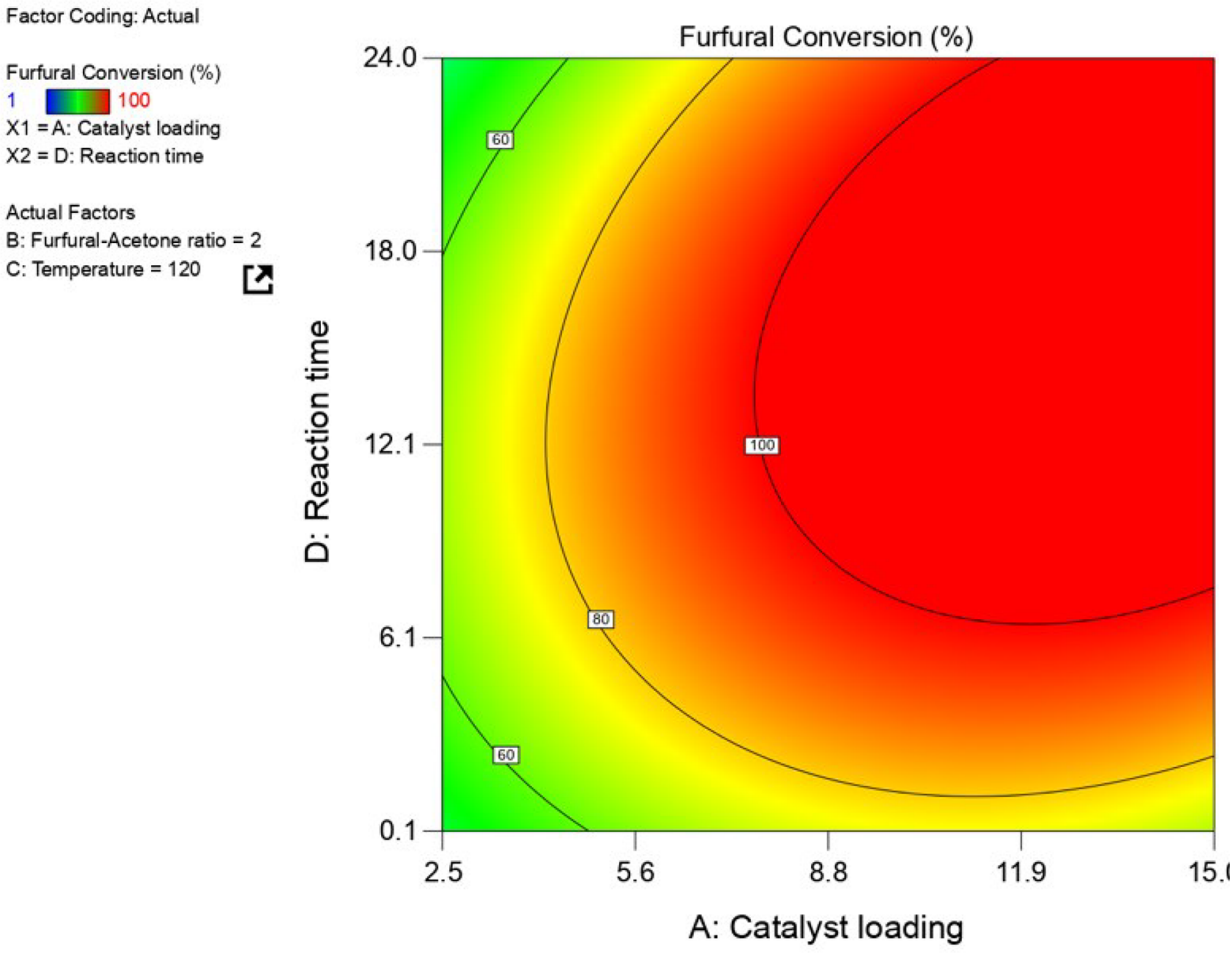
Contour plot showing the furfural conversion (%) versus the catalyst loading and the reaction time at a constant reaction temperature of 120 °C and a furfural/acetone ratio of 1.95 (mol/mol).

Contour plots showing the effects of the catalyst loading and batch time on the molar yield of the C_13_ product, furfural conversion, and mass yield of solids, are shown in Figures 6 and 7 and Figure S8 of the Supporting Information, respectively [all at a fixed temperature of 120 °C and furfural/acetone molar ratio in the feed of 1.9 (mol/mol)]. The conversion of furfural sharply increases with the mass of catalyst used, even at very short reaction times. The yields of C_13_ also follow a trend similar to the furfural conversion but decrease at extreme conditions of high catalyst loading and reaction time. On the other hand, the mass of solids produced also shows a sharp increase at these severe reaction conditions. Hence, at the more severe conditions of long reaction times, high catalyst loading, and also higher reaction temperature, the desired products are not stable and further condensation reactions occur, leading to the formation of solids.

**Figure 8:**
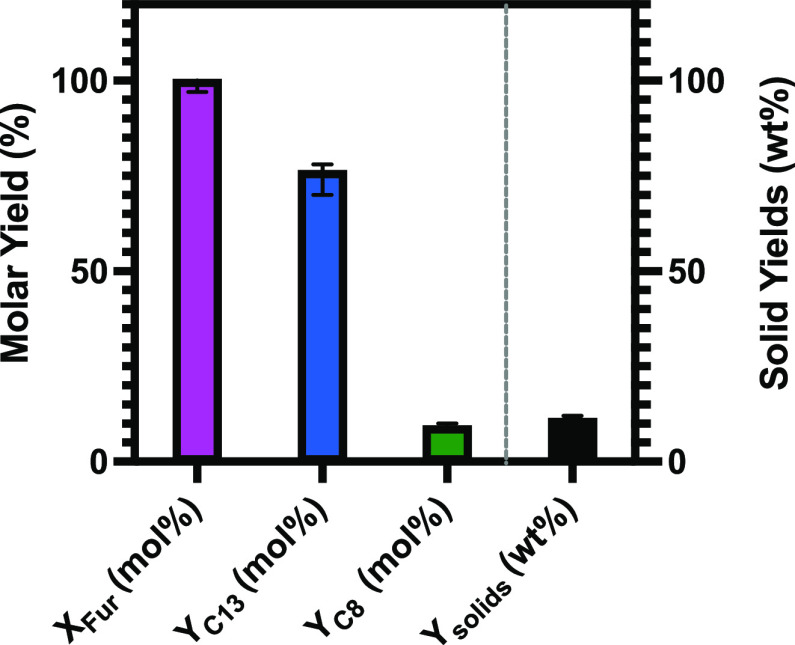
Results of the optimization experiments of the cross-aldol condensation of furfural and acetone. Process conditions: temperature, 120 °C; catalyst loading, 6.9 (wt %); furfural/acetone ratio, 1.8 (mol/mol); and batch time, 9 h.

From the results of the DOE, regions of process conditions where the yield of C_13_ product is highest were identified. Additional experiments were performed at these modeled optima, and the highest product yield of C_13_ within the given operating window is shown in Figure 8 [120 °C, catalyst loading of 6.9 (wt %), furfural/acetone ratio of 1.8 (mol/mol), and batch time of 9 h]. Furfural conversion was about 100%, and the preferred C_13_ product was obtained at a molar yield of 78 mol %. The molar yield of the C_8_ product was 8 mol %, whereas the solid yield was below 10 wt %. The overall carbon yield for the aldol condensation step at optimized conditions was calculated to be around 90%.

### Catalytic Hydrotreatment

**Figure 9:**
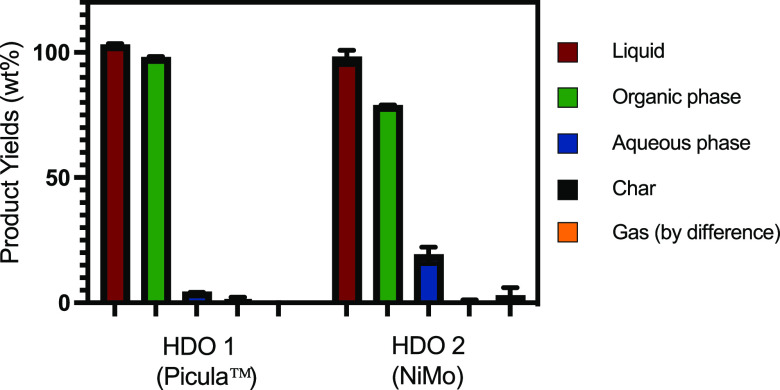
Individual product yields from the catalytic hydrotreatment experiments. Process conditions: HDO 1, temperature, 175–275 °C; pressure, 100 bar; and batch time, 4 h; HDO 2, temperature, 350 °C; pressure, 150 bar; and batch time, 4 h.

The liquid products of the cross-aldol condensation reactions were deoxygenated to liquid hydrocarbons in a two-step catalytic hydrotreatment process at two different temperatures in a batch setup. The aim of the first low-temperature step (175–275 °C) is to hydrogenate the reactive groups in the C_13_ product (ketone and C–C double bonds) to compounds that are less prone to condensation reactions, thereby limiting char formation. For this purpose, a Ni-based Picula catalyst was used, which has shown to be very active for aldehyde/ketone hydrogenation to alcohols.^48,49^ The products of the first hydrotreatment step were isolated and used for a second hydrotreatment step, with a NiMo catalyst at higher temperatures, with the intention to obtain hydrocarbons with a low level of oxygenates. The product yields after the hydrotreating steps are shown in Figure 9. Liquid yields were very promising (>95 wt %), and hardly any char formation was detected (<1 wt %). The gas yields were also minimal, with a maximum of 4 wt % measured for the second HDO step with NiMo. The liquid products obtained after both steps were biphasic, with a lighter organic phase on the top and an aqueous phase on the bottom. The elemental composition of the organic phases of both steps were measured using elemental analysis. The results are listed in Table 3, along with the elemental composition of the feed as a reference.

**Table 3:** Elemental Composition of the Products from Catalytic Hydrotreatment Experiments

	cross-aldol condensed products	after HDO 1	after HDO 2
C (wt %)	71.71	71.63	85.84
H (wt %)	6.02	11.53	12.48
O (wt %, difference)	22.20	16.84	1.68
atomic H/C	1.01	1.93	1.75
atomic O/C	0.23	0.18	0.01

A sharp increase in the H/C ratio is seen in the product after the first HDO step as a result of hydrogenation reactions, e.g., of the ketone fragment and C–C double bonds. However, the products after the first HDO step still contain a significant amount of oxygen (17 wt %), indicating that deep deoxygenation is not possible at these conditions (275 °C). Elemental analysis of the product after the second HDO using NiMo at elevated temperatures shows an oxygen content of 1.68 wt %, indicating that the hydrogenolysis of ether bonds in hydrogenated furans and are easily cleaved at these conditions. In addition, the C content has increased significantly (Table 3), and the H/C ratio is lowered. The latter is among others due to the formation of aromatics (*vide infra*), which have a lower H/C ratio than saturated hydrocarbons. The calculated carbon yields for each HDO step are high and above 97%. Thus, a two-step catalytic hydrotreatment is hence very efficient to achieve significant levels of oxygen removal in combination with high carbon yields.

The liquid products obtained after each of the hydrotreatment steps were analyzed using ^13^C NMR, GC–MS, and GC × GC/TOF-MS analyses.

**Figure 10:**
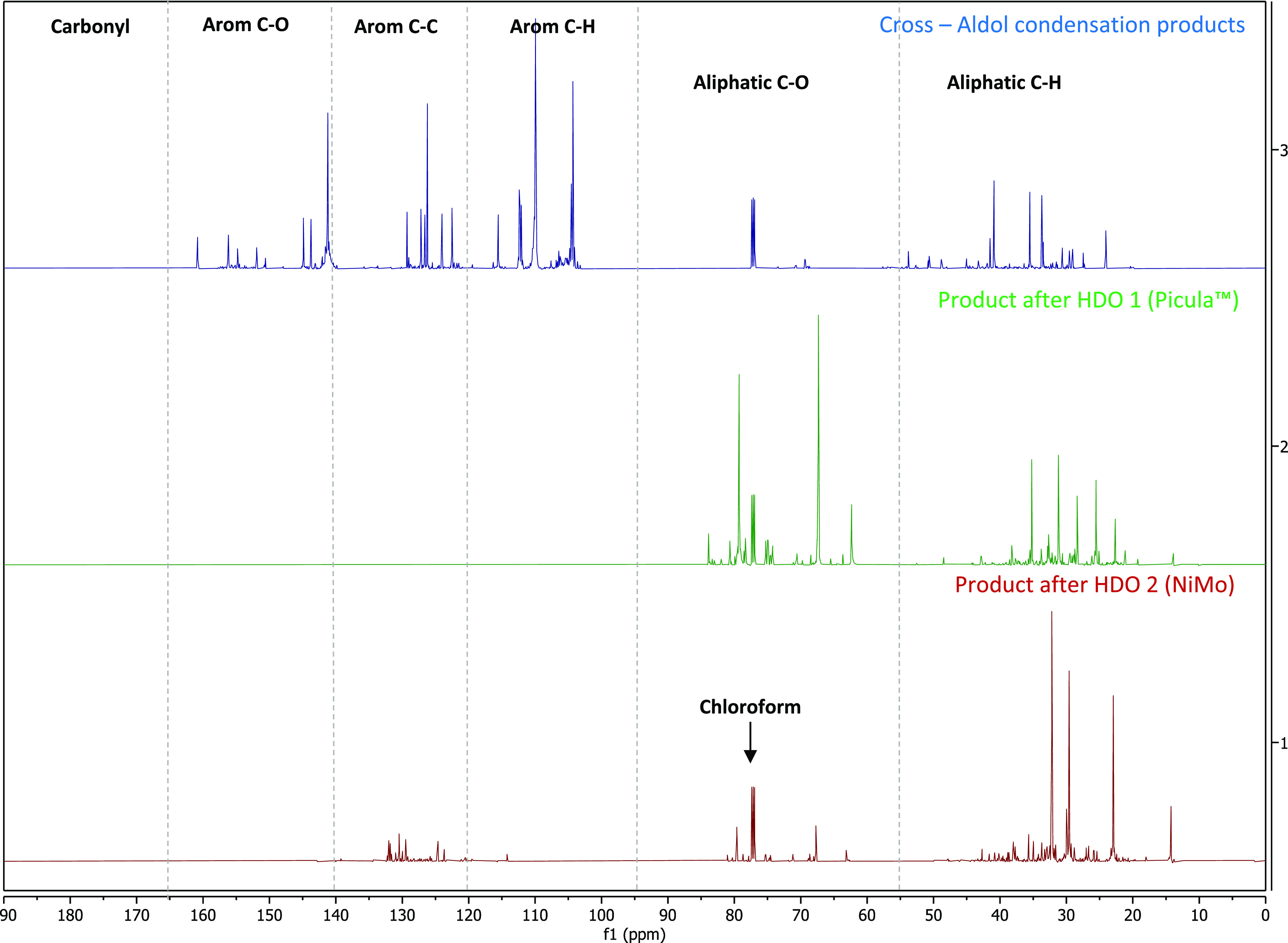
^13^C NMR spectra of (top) products from the cross-aldol condensation, (middle) liquid product after HDO 1, and (bottom) final liquid product after HDO 2.

^13^C NMR spectra of the products from the two HDO steps and the products of the cross-aldol condensation reactions were recorded and show substantial differences (Figure 10). The C=C linkages of the furan units (δ = 120–140 ppm) appear to be completely hydrogenated to aliphatic C–H linkages (δ = 0–55 ppm) after the first HDO step 1. The ketonic C=O linkages (δ = 140–165 ppm) have also disappeared. The final product after catalytic hydrotreatment is composed mainly of aliphatic C–H units and a small amount of aromatic C–H units.

GC–MS chromatograms (Figure S10 of the Supporting Information) show that the C_8_ product (peak 1) and C_13_ product (peak 2) are deoxygenated to C_8_ (peak 7) and C_13_ (peak 10) alkanes during the two-step hydrotreatment process. Besides, other saturated cycloalkanes were also visible in the final product, indicating hydrocracking and molecular rearrangements during hydrotreatment.

**Figure 11:**
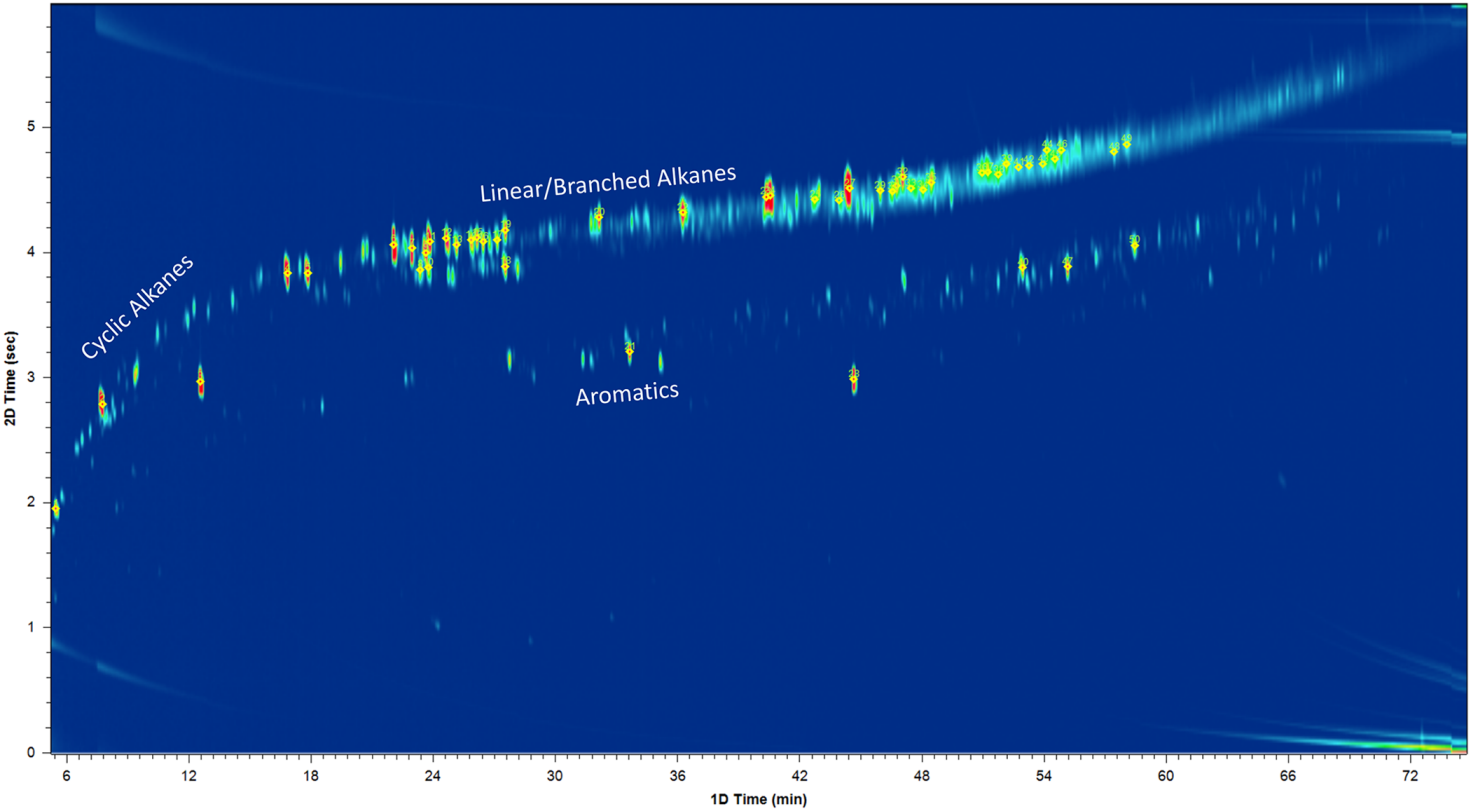
GC × GC/TOF-MS analysis results of the final liquid product after catalytic hydrotreatment.

To obtain a better understanding of the various product groups in the final hydrotreated product, GC × GC/TOF-MS analysis was performed. The chromatogram of the final product is shown in Figure 11, and a list with the 20 most abundant compounds identified is shown in Table S6 of the Supporting Information. Only three major product classes were identified: straight-chain/branched alkanes, saturated cycloalkanes, and aromatic hydrocarbons. Hence, the final product is rich in hydrocarbons, with a low number of oxygenated compounds identified, in line with the elemental analysis data (*vide supra*Table 2).

**Figure 12:**
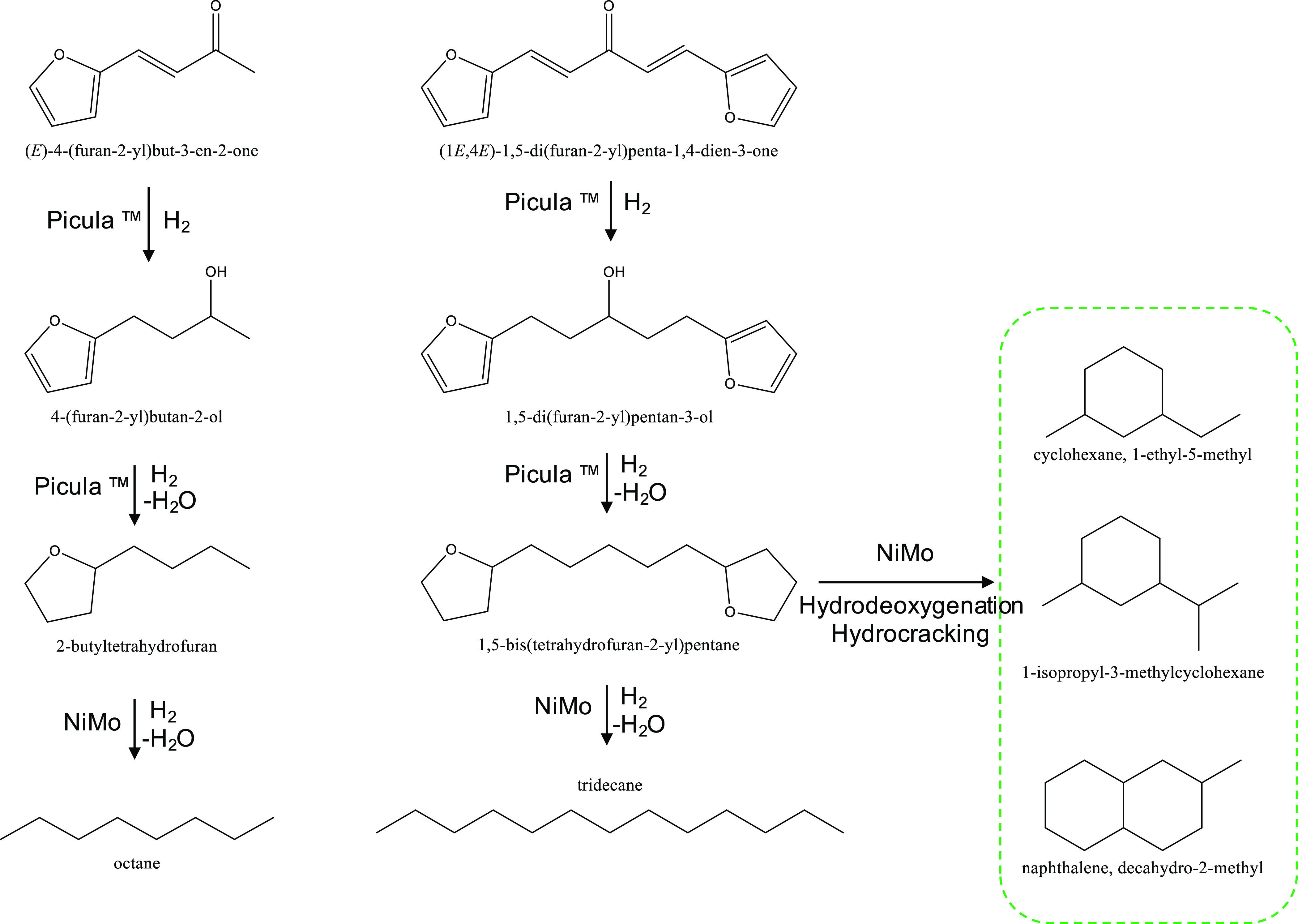
Proposed reaction sequence for the hydrodeoxygenation of the cross-aldol condensation products of furfural and acetone.

On the basis of the analysis of the feed and hydrotreated product and literature precedents,^33,37,40−43,50^ a reaction network is proposed for the two hydrotreatment steps (Figure 12). The aliphatic C=C bonds and the ketone fragments in the C_8_ and C_13_ products (C=O bond) are initially hydrogenated by the Picula catalyst. Subsequently, the −OH group is removed as water, and fully hydrogenated products (e.g., *n*-butyltetrahydrofuran from the C_8_ product and 1,5-bis(tetrahydrofuran-2-yl)pentane from the C_13_ product) are formed. These are prone to hydrogenolysis reactions and deoxygenation at elevated temperatures and pressures using NiMo (HDO 2 step) giving the corresponding alkanes (octane and tridecane) or hydrocracking/rearrangement products thereof.

### Overall Carbon Yields for the Conversion of Wood to Middle Distillates

**Figure 13:**
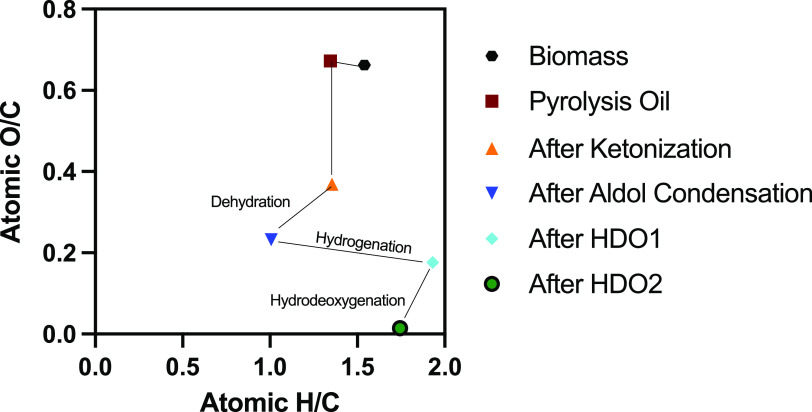
van Krevelen plot of the conversion of biomass to hydrocarbons using the approach discussed in the text. The catalytic upgrading steps of ketonization, aldol condensation, and catalytic hydrotreatment were studied using furfural and acetic acid and not a representative pyrolysis oil.

The elemental composition of the various products within the given concept is shown in the form of a van Krevelen plot in Figure 13. The original biomass has a H/C molar ratio of 1.54 and an O/C ratio of 0.66 (dry basis). Upon pyrolysis in molten salt, the ratios hardly changed as a result of the formation of large amounts of furfural and acetic acid, with elemental compositions close to that of the biomass source. The ketonization reaction of acetic acid is accompanied by decarboxylation and dehydration, as evidenced by a drop in the O/C ratio. Cross condensation of the formed acetone and furfural leads to water formation, and this is clearly shown in the reduction of both the H/C and O/C ratios after the cross-aldol condensation step. Another interesting observation from the van Krevelen plot is that the H/C ratio increases significantly during the first catalytic hydrotreatment step with Picula, indicating the occurrence of hydrogenation reactions, in line with the GC and NMR data. In the second hydrotreatment step, most oxygen is removed, primarily as water (dehydration), to arrive at a final product with a very low O/C ratio.

**Figure 14:**
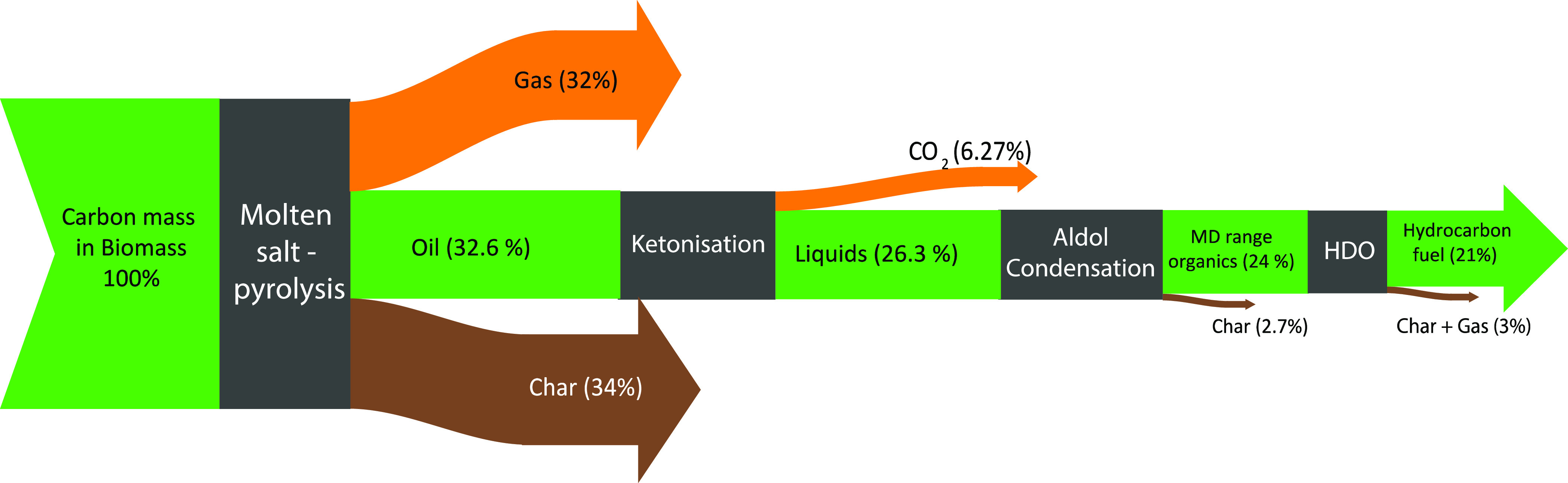
Carbon flow of the proposed concept for producing hydrocarbons from pinewood biomass in molten salt. The catalytic upgrading steps of ketonization, aldol condensation, and catalytic hydrotreatment were studied using furfural and acetic acid and not a representative pyrolysis oil.

On the basis of the experimental results and product analysis after each of the individual steps in the value chain, from pinewood biomass to hydrocarbon fuel, an overall carbon efficiency can be established. The carbon flow diagram or Sankey diagram visualizes the major losses in the proposed value chain and visualizes the overall carbon yield of the process (see Figure 14). Most carbon losses are in the molten salt pyrolysis of pinewood, where a third of input carbon is lost as char and another third is lost as gaseous components.

The loss of carbon is minimal in the subsequent catalytic conversion steps (ketonization, cross-aldol condensation, and hydrotreatment). The ketonization of acetic acid to acetone stoichiometrically releases 1 mol of carbon as CO_2_, which accounts for a carbon loss of 6.2%. Cross-aldol condensation of furfural and acetone was shown to be highly selective to the preferred C_13_ product. Solids formation was minimized by optimization of the process conditions to less than 2.7%. In the two-step HDO process, coke formation was very limited. However, a small fraction of carbon (2.5%) was lost as volatile hydrocarbons and non-condensable gases (CO and CO_2_).

On the basis of these findings, an overall carbon yield of 21%, from dry biomass to a hydrocarbon-rich fuel, was calculated. Here, it is assumed that the separation of the main components in the oil after pyrolysis in molten salts (furfural and acetic acid) is quantitative and without carbon losses [e.g., by (vacuum) distillation].

As a comparative benchmark, the integrated hydropyrolysis and hydroconversion technology (IH^2^), developed by the Gas Technology Institute (GTI) to produce gasoline- and diesel-grade fuel from woody biomass, reported an overall liquid yield in the range of 25–28 wt %, which also includes a significant fraction of lighter hydrocarbons, such as butane.^51,52^ When translated to carbon yields, the overall yield reported is around 40–46%. Although the carbon yield demonstrated in this study is lower than that of the IH^2^ process, the major source of carbon loss in this study is from the pyrolysis step in molten salt. Optimization and the use of pressured hydropyrolysis instead of atmospheric pyrolysis could potentially increase the liquid yields and improve carbon yields.

## Conclusion

A novel process concept to obtain a hydrocarbon-rich product with a low oxygen content from pinewood biomass involving molten salt pyrolysis followed by separation and further catalytic conversions of isolated furfural and acetic acid was investigated. Molten salts were used to decompose pinewood to a liquid mixture composed almost entirely of furfural and acetic acid. To reduce complexity and experimental issues related to the production of sufficient amounts of pyrolysis oils, further catalytic upgrading was studied in detail, with representative components from the pyrolysis oil. Acetic acid dissolved in water was converted to acetone using a ketonization approach in a continuous fixed bed reactor containing a CeZrO_*x*_ catalyst. The ceria catalyst used was shown to be very stable, and acetic acid conversion was complete for a runtime of at least 230 h. Obtained acetone and furfural were coupled using a cross-aldol condensation reaction using a Mg/Al hydrotalcite catalyst. The process conditions of the cross-aldol condensation reaction were optimized using a DOE to maximize the yield of the C_13_ product at full conversion of furfural. The products from the cross-aldol condensation step were finally deoxygenated by a two-step hydrotreatment approach using Picula and NiMo catalysts to obtain a hydrocarbon-rich final product with an oxygen content of 1.68 wt %. An overall carbon yield of 21% was calculated for the overall concept, with the assumption that separation of acetic acid and furfural after molten salt pyrolysis is quantitative.

## Supporting Information Available

Elemental composition of the biomass used, description of the experimental setup, ketonization experiments at full conversion, ketonization experiments using simulated pyrolysis oils, statistical modeling information and results, FTIR and GC chromatograms, and product identification (PDF)
