# Supplementary material for: Novel Route to Produce Hydrocarbons from Woody Biomass Using Molten Salts
Source: Energy Fuels. 2022 Oct 11;36(20):12628–40. doi: 10.1021/acs.energyfuels.2c02044 (PMC9589755; doi:10.1021/acs.energyfuels.2c02044)
Supplement: Supplementary file 1 — ef2c02044_si_001.pdf [file ef2c02044_si_001.pdf]

Supplementary Information for:

A novel route to produce hydrocarbons from woody biomass using molten salts

Balaji Sridharan[a], Homer C. Genuino[a], Daniela Jardan[a], Erwin Wilbers[a], Henk H. van de Bovenkamp [a], Jozef G. M. Winkelman[a], Robbie H Venderbosch[b], Hero J Heeres[a*]

[a] Department Chemical Engineering, Engineering and Technology institute Groningen (ENTEG), University of Groningen, Nijenborgh 4, Groningen, 9747 AG, The Netherlands

[b] Biomass Technology Group B.V., Josink Esweg 34, 7545 PN Enschede, The Netherlands

*Corresponding author (h.j.heeres@rug.nl)

Table S1: Elemental composition of the pinewood biomass used in this study

**Ultimate analysis wt% d.b.**		
**Carbon**	49.61	wt%
**Hydrogen**	6.37	wt%
**Nitrogen**	0.1	wt%
**Sulfur**	0.1	wt%
**Oxygen**	43.82	wt%
**LHV**	19.76	MJ/kg
**Moisture**	5	wt%

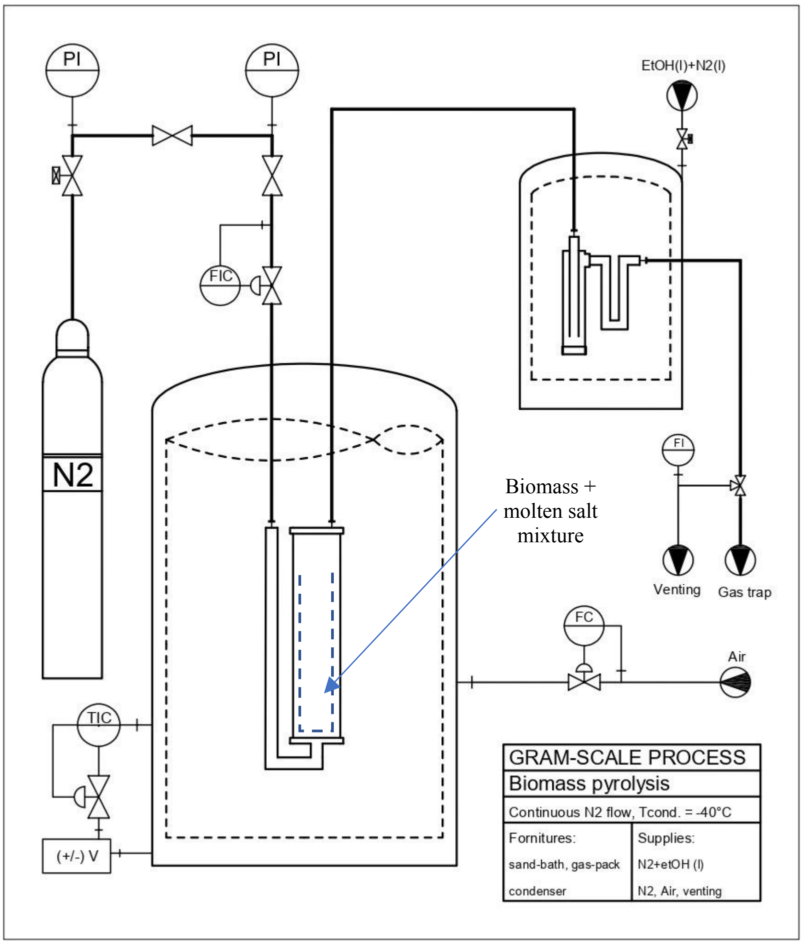

Figure S1: Schematic of the gram scale pyrolysis reactor used

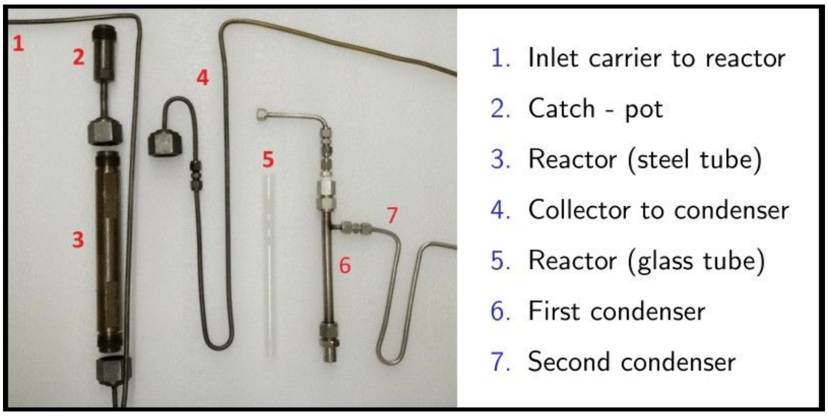

Figure S2: Picture of the gram scale pyrolysis reactor used

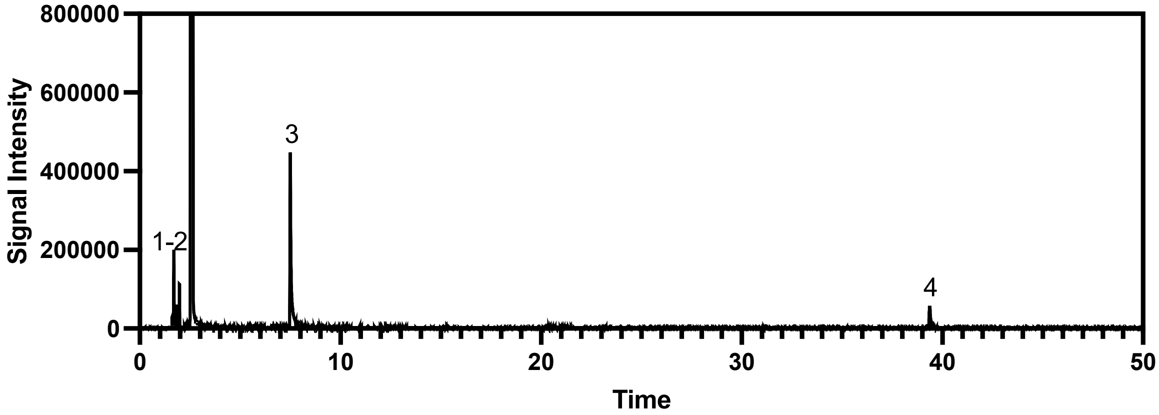

Figure S3: GC-MS chromatogram of the pyrolysis oil obtained from molten salt pyrolysis of pinewood. Compounds identified: 1- Impurity (ethanol from the condenser system), 2-Acetic Acid, 3- Furfural and 4- butylated hydroxytoluene (stabilizer of the THF solvent used)

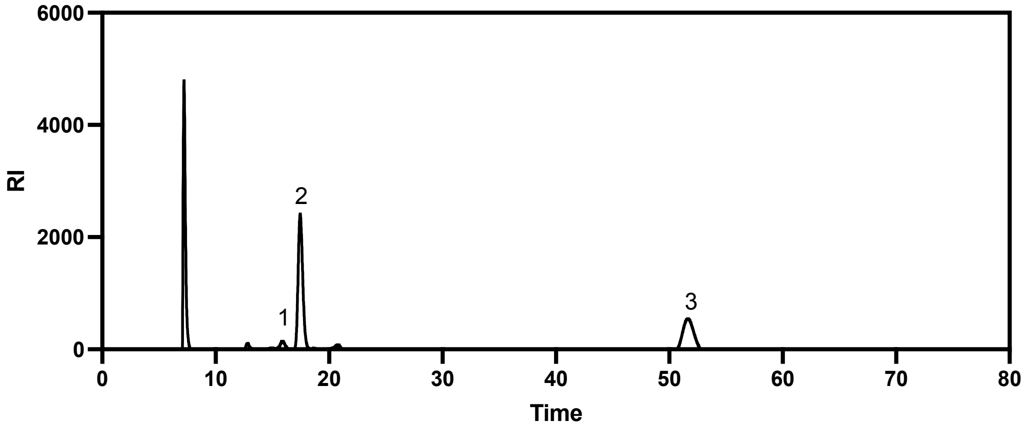

Figure S4: HPLC chromatogram of the pyrolysis oil obtained from molten salt pyrolysis of pinewood. Compounds identified: 1- Formic Acid, 2-Acetic Acid, 3- Furfural

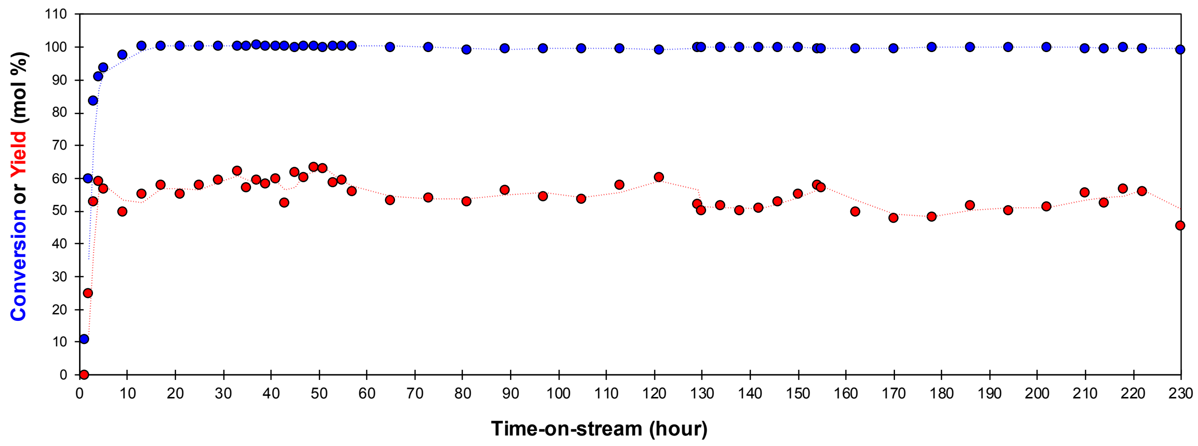

Figure S5: Conversion of acetic acid and corresponding yield of acetone from the continuous ketonization experiments. Process conditions: 10 wt.% acetic acid in water, 2.4 mL min^{-1}, 1.0 g catalyst, 300 °C (conversion: blue dots, yield: red dots)

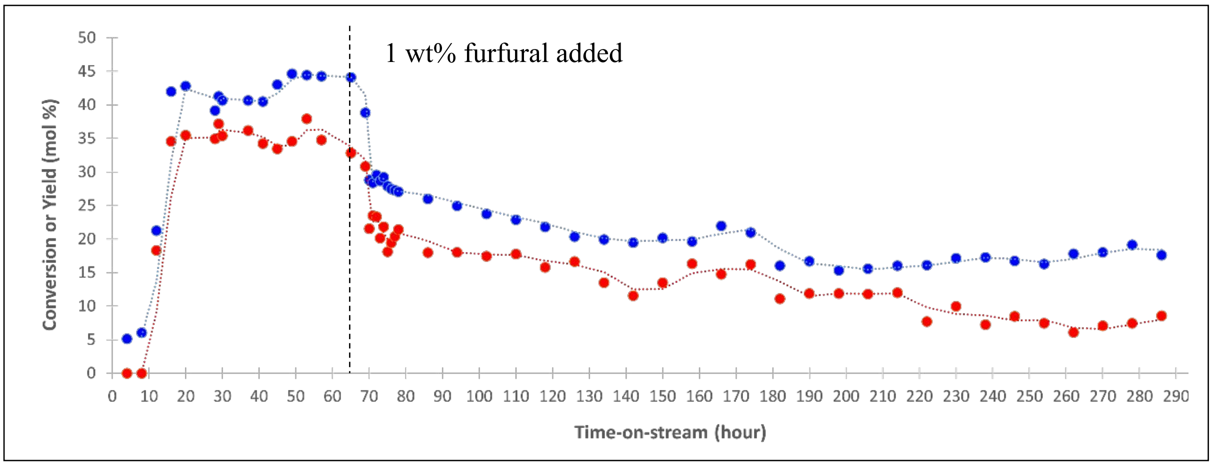

Figure S6: Influence of furfural on the ketonization of acetic acid (10 wt.% acetic acid in water, 1 wt.% furfural; 2.4 mL h^{-1}, 1.0 g $CeZrO_x$, 250 °C, 1 atm, conversion: blue dots, yield: red dots).

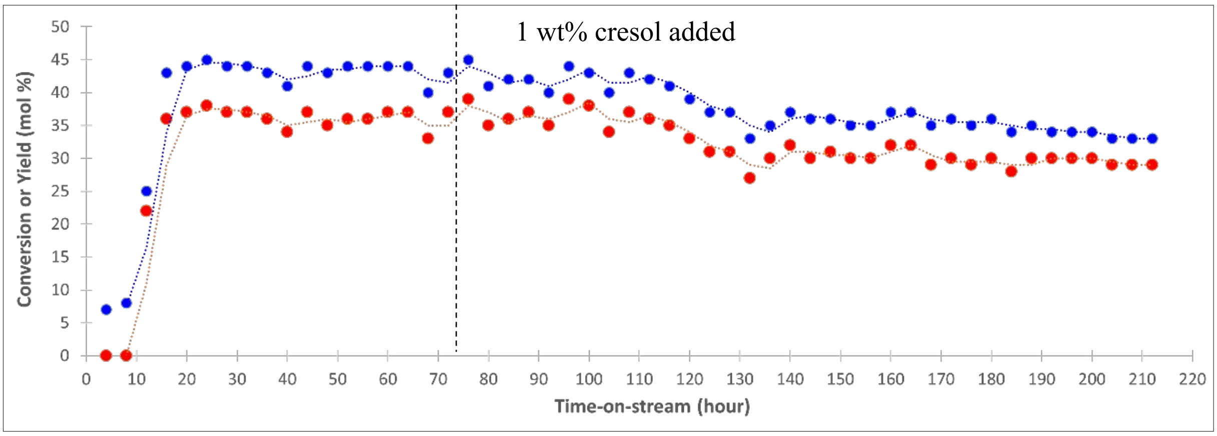

Figure S7: Influence of p-cresol on the ketonization of acetic acid (10 wt.% acetic acid in water, 1 wt.% p-cresol; 2.4 mL h^{-1}, 1.0 g $CeZrO_x$, 250 °C, 1 atm)

Table S2: Results of the DOE used to optimize the cross- aldol condensation reaction

Run	A:Catalyst Loading	B:Furfural-Acetone Ratio	C:Temperature	D:Reaction Time	C8 Yield	C13 Yield	Solid Yield	Furfural Conversion
	(wt%)	(mol/mol)	°C	Hours	mol%	mol%	mass%	%
**1**	6.94	1.75	120	9.0	7.10	72.70	11.90	100
**2**	5.88	2.01	50	24.0	0.00	0.00	0.80	0.8
**3**	15.00	2.50	120	0.2	3.10	52.70	13.10	76
**4**	6.25	0.50	118	24.0	0.00	0.00	8.70	100
**5**	15.00	1.92	115	24.0	2.03	57.70	21.50	100
**6**	5.67	2.50	93	0.2	0.00	0.00	0.80	2
**7**	9.94	2.50	83	14.1	9.00	24.30	1.70	40
**8**	15.00	0.50	115	6.9	0.00	0.00	16.10	100
**9**	6.94	1.75	120	9.0	8.00	78.50	9.70	97
**10**	15.00	0.50	50	1.5	10.00	8.00	1.70	28
**11**	2.50	2.50	120	24.0	6.90	10.90	0.80	30
**12**	15.00	0.50	64	24.0	0.00	2.70	16.00	100
**13**	5.94	0.50	50	6.1	1.10	0.00	0.50	5
**14**	2.50	2.50	50	0.2	0.00	0.00	0.20	1
**15**	2.50	1.26	82	15.1	0.00	0.00	0.40	1
**16**	15.00	2.35	50	24.0	6.70	11.50	2.40	24
**17**	9.92	1.33	83	0.2	2.60	0.70	1.00	8
**18**	2.50	1.26	82	15.1	0.00	0.00	0.50	1
**19**	15.00	1.99	50	6.2	3.20	1.90	1.90	10
**20**	2.50	0.50	120	0.2	0.00	0.00	0.50	2

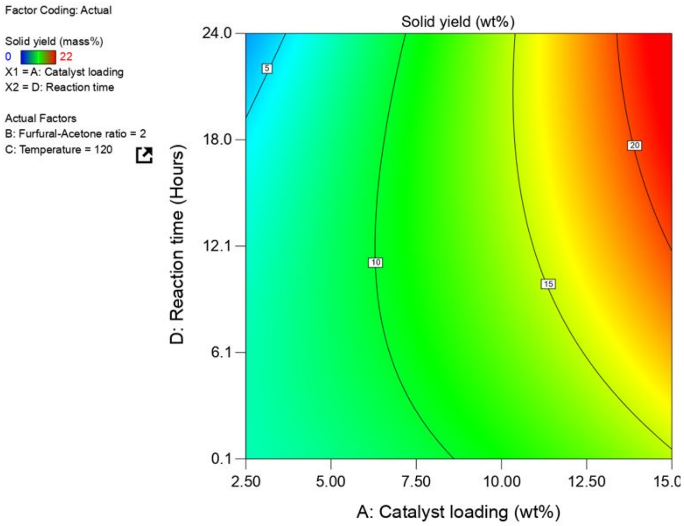

Figure S8: Contour plot showing the variation of the solid mass yield on catalyst loading and reaction time at a constant reaction temperature of 120C and a feed with furfural-acetone ratio of 1.9 (mol/mol)

S1.1 Statistical Modelling

Regression analysis were performed to establish the dependence of the C_{13} molar yield, furfural conversion and solid mass yield with four process variables viz: the furfural-acetone molar ratio, reaction temperature, batch time and catalyst loading. Analysis of the data using design expert software showed that a quadratic model is the best representation for each of the three responses with a reasonably good fit ($R^2 > 87\%$). A summary of the models and the corresponding model equations for each of the responses are shown in equations S1 – S3 and Table S3.

$$C_{13}\,Yield\,(mol\%) = 19.71 + 8.45 * A + 10.36 * B + 16.45 * C - 1.04 * D + 12.35 * AD + 13.89 * BC - 18.5 * B^2 + 0.01 * C^2 + 0.01 * D^2 \quad (S1)$$

$$Furfural\,Conversion\,(\%) = -3.74 + 13.72 * A + 32.31 * B - 3.27 * C + 6.71 * D - 2.41 * AB + 0.14 * AD - 1.67 * BD - 0.40 * A^2 + 0.02 * C^2 - 0.16 * D^2 \quad (S2)$$

$$Solid\,Yield\,(wt\%) = 5.09 + 4.97 * A - 1.82 * B + 4.66 * C + 2.19 * D - 1.74 * AB + 2.77 * AC - 2.8 * AD + 1.27 * BC - 2.12 * BD - 2.84 * B^2 + 3.46 * C^2 \quad (S3)$$

Where,

A = Catalyst Loading (wt/wt %)

B = Furfural – Acetone ratio (mol/mol)

C = Reaction Temperature (°C)

D -Reaction time (hours)

Table S3: DOE model summary

Source	C_{13} Dimer Yield (mol%)			Furfural Conversion (%)			Solid Yield (wt%)		
	**VIF**	**p-value**	**R^2**	**VIF**	**p-value**	**R^2**	**VIF**	**p-value**	**R^2**
**Model**	-	0.0031	0.863	-	< 0.0001	0.9671	-	< 0.0001	0.9866
A-Catalyst loading	1.13	0.0272		1.11	< 0.0001		1.15	< 0.0001	
B-Furfural-Acetone ratio	1.06	0.0191		1.06	0.022		1.11	0.0011	
C-Temperature	1.05	0.001		1.09	< 0.0001		1.11	< 0.0001	
D-Reaction time	1.06	0.846		1.05	0.0004		1.11	0.0002	
AB				1.09	0.004		1.17	0.0027	
AC							1.19	0.0002	
AD	1.23	0.0219		1.14	0.0283		1.26	0.0002	
BC	1.18	0.0112					1.21	0.0104	
BD				1.18	0.0007		1.3	0.0008	
A^2	1.13	0.165		1.14	0.0326		1.15	0.2098	
B^2	1.31	0.0408					1.4	0.0064	
C^2	1.13	0.0073		1.19	0.0013		1.24	0.0008	
D^2	1.37	0.0798		1.22	0.0061		1.39	0.0752	

The p-valuea of all three models and most of the terms considered in the model are less than 0.05, suggesting that they are statistically significant. The VIF value of most of the terms is less than 1.3, indicating limited correlation between the terms. The reasonably high R^2 values for all three models indicate that the models accurately represent the experimental results obtained.

FT-IR analysis results of the liquid products obtained after each of the hydrotreatment step were compared with that of the cross-aldol condensed products and the results are given in

Figure S9. In the hydrogenation step of the catalytic hydrotreatment, the stretch due to the ketonic C=O linkage completely disappears, as evidenced by the absence of the peak at 1225 cm^{-1}. The IR spectra of the product after hydrogenation have only 4 major peaks which correspond to the alkyl C-H chains (at 2800 – 3000 cm^{-1} and 1450 cm^{-1}) and C–O chain in the furan structure (1210 – 1233 cm^{-1}). This observation is in line with the results of the elemental analysis, where all furans were shown to be strongly hydrogenated. The final product after the catalytic hydrotreatment shows only the presence of the alkyl C-H chains while the peak corresponding to the C-O chain is completely absent.

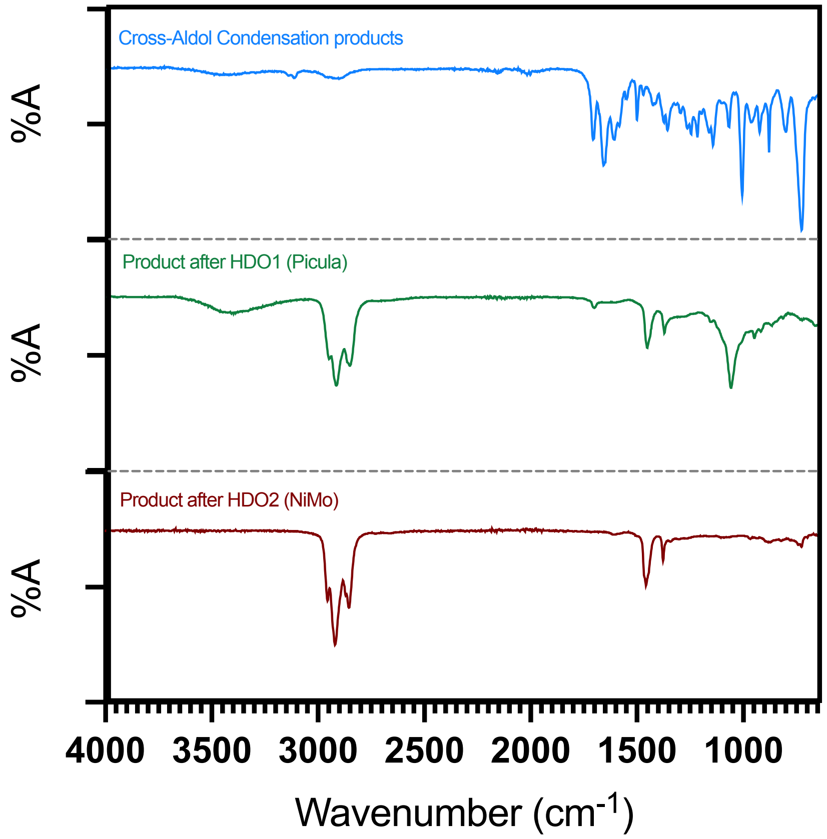

Figure S9: FT-IR analysis results of the products from cross-aldol condensation (top), of the liquid product after hydrogenation (HDO1) (middle) and of the final liquid product after hydrodeoxygenation (HDO2) (bottom).

Table S4: Peak assignments for the FTIR spectrum. Adapted from [1-4]

Label	Wavenumber (cm^{-1})	Assignment	Functional groups and structures in lignin
1	3400-3600	O-H	Free -OH
1	3100-3400	O-H	Associated -OH
2	2820-2960	C-H	$-CH_2$, $-CH_3$
2	2920	C-H	Carboxylic -OH
3	2650-2890	C-H	Methyl group in $–OCH_3$
4	1700-1800	C=O	Unconjugated ketones, carbonyls and esters
5	1650-1680	C=O	Conjugated *p*-substituted carbonyl and carboxyl
6	1500-1600	Arom. skeletal	Benzene rings; C=C stretch in furans
7	1450-1470	C-H	Asymmetric vibrations in $-CH_2$, $-CH_3$
8	1300-1400	Arom. skeletal	Benzene rings; C-H in-plane deformation
9	1270-1290	C-O	Guaiacyl
10	1214-1233	C-O	C-C plus C-O plus C=O strech
11	1140-1145	C-H	Guaiacyl
12	1000-1100	C-H, C-O	Aromatic ring; C-O in alcohols and ethers
13	900-920	C-H	Aromatic ring
14	850-860	C-H	C-H out-of-plane in positions 2, 5 and 6 of guaiacyl

Table S5: 13C-NMR integration regions

Group	δ range (ppm)
Aliphatics	0-55
Methoxy groups	55-57
Aliphatic C-O	57-95
Aromatic C-H	95-122
Aromatic C-C	122-139
Aromatic C-O	139-165

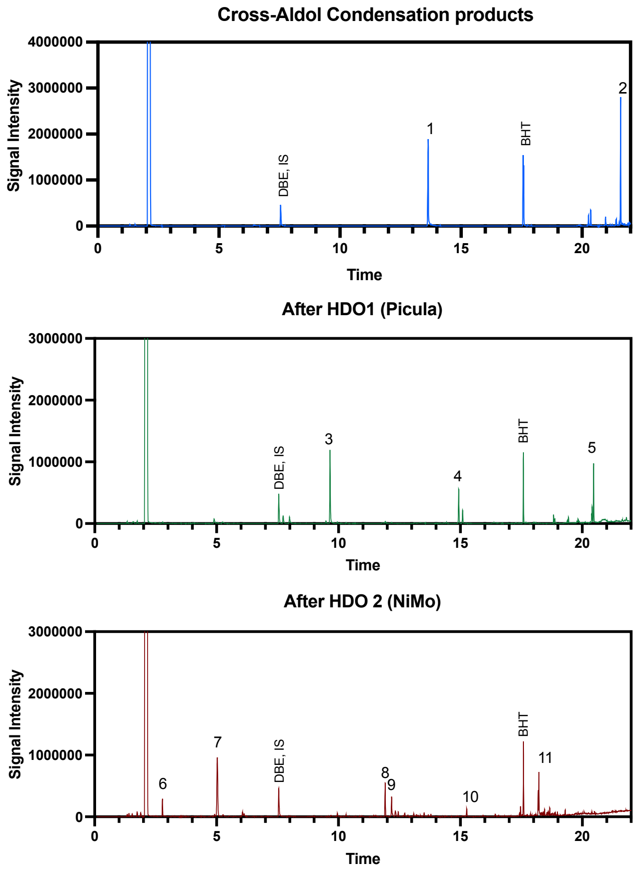

Peak	ID	Structure
1	3-Buten-2-one, 4-(furanyl)-	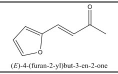
2	1,5-Di(furan-2-yl)penta-1,4-dien-3-one	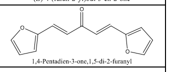
3	2-Butyltetrahydrofuran	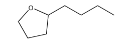
4	2-(5-methylhexyl)tetrahydrofuran	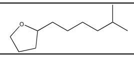
5	1,5-bis(tetrahydrofuran-2-yl)pentane	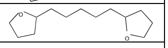
6	Heptane	
7	Octane	
8	Cyclohexane, 1-ethyl-5-methyl-	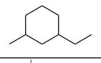
9	Cyclohexane, 1-methyl-3-(1-methylethyl)-	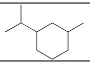
10	Tridecane	
11	Unknown	

Figure S10: GC-MS chromatogram of the products from cross-aldol condensation (top), liquid product after HDO1 (middle) and final liquid product after HDO2 (bottom). Compounds identified are lister on the table on the right.

Table S6: List of compounds identified from the 2DGC ToF-MS analysis

Peak(#)	Compound	RT1(min)	RT2(s)
6	Cyclohexane, 1-methyl-4-(1-methylethyl)-, trans-	22.06	4.06
27	1,7-Dimethyl-4-(1-methylethyl)cyclodecane	44.46	4.51
24	1,7-Dimethyl-4-(1-methylethyl)cyclodecane	40.56	4.45
7	Cyclohexane, 1-methyl-4-(1-methylethyl)-, cis-	22.96	4.03
3	*n-Butyl ether – Internal Standard*	12.56	2.96
2	Octane	7.76	2.78
28	*Butylated Hydroxytoluene - Stabilizer*	44.66	2.98
4	Cyclohexane, 1-methyl-4-(1-methylethyl)-, trans-	16.86	3.82
23	Cyclohexane, 1,2-dimethyl-3-pentyl-4-propyl-	40.36	4.44
11	Naphthalene, decahydro-2-methyl-	23.86	4.08
22	Cyclohexane, 1,5-diisopropyl-2,3-dimethyl-	36.26	4.31
5	Cyclohexane, 1-methyl-4-(1-methylethyl)-, trans-	17.86	3.83
9	cis-Decalin, 2-syn-methyl-	23.66	3.99
37	1H-Indene, 5-butyl-6-hexyloctahydro-	51.26	4.64
12	Naphthalene, decahydro-2-methyl-	24.66	4.11
32	Naphthalene, 2-decyldecahydro-	47.06	4.59
31	Naphthalene, 2-decyldecahydro-	46.76	4.53
45	1H-Indene, 5-butyl-6-hexyloctahydro-	54.56	4.74
35	Naphthalene, 2-decyldecahydro-	48.46	4.55
18	Naphthalene, 1,2,3,4,4a,5,6,7-octahydro-4a-methyl-	27.56	3.88
20	Heptadecane	32.16	4.27

[1] B. Scholze, D. Meier, Characterization of the water-insoluble fraction from pyrolysis oil (pyrolytic lignin). Part I. PY–GC/MS, FTIR, and functional groups, Journal of Analytical and Applied Pyrolysis. 60 (2001) 41–54. doi:10.1016/S0165-2370(00)00110-8.

[2] S. Wang, H. Lin, B. Ru, W. Sun, Y. Wang, Z. Luo, Comparison of the pyrolysis behavior of pyrolytic lignin and milled wood lignin by using TG–FTIR analysis, Journal of Analytical and Applied Pyrolysis. 108 (2014) 78–85. doi:10.1016/j.jaap.2014.05.014.

[3] J. Chen, C. Liu, S. Wu, J. Liang, M. Lei, Enhancing the quality of bio-oil from catalytic pyrolysis of kraft black liquor lignin, RSC Adv. 6 (2016) 107970–107976. doi:10.1039/C6RA18923G.

[4] Y. Lu, Y.-C. Lu, H.-Q. Hu, F.-J. Xie, X.-Y. Wei, X. Fan, Structural Characterization of Lignin and Its Degradation Products with Spectroscopic Methods, Journal of Spectroscopy. (2017). doi:10.1155/2017/8951658.
